# CRISPR/Cas9‐Mediated Knockout of *LbSP1* and *LbSP5G1* Reveals Efficient Customization of Shoot Architecture in Black Goji Berry

**DOI:** 10.1111/pbi.70753

**Published:** 2026-09-02

**Authors:** Fazal Rehman, Huanfang Liu, Yun Ma, Shaohua Zeng, Yan Wu, Yuan Zong, Chao Yang, Ying Wang

**Affiliations:** ^1^ State Key Laboratory of Plant Diversity and Specialty Crops, Guangdong Provincial Key Laboratory of Digital Botanical Garden, Key Laboratory of National Forestry and Grassland Administration on Plant Conservation and Utilization in Southern China, South China Botanical Garden, Chinese Academy of Sciences, South China National Botanical Garden Guangzhou China; ^2^ College of Advanced Agricultural Sciences University of Chinese Academy of Sciences Beijing China; ^3^ College of Life Science Gannan Normal University Ganzhou China; ^4^ School of Chinese Medicine Hong Kong Baptist University Hong Kong China

**Keywords:** CRISPR/Cas9, determinate, goji berry, indeterminate, mechanical harvesting, protein–protein, *SP*/*SP5G*, WGCNA

## Abstract

Goji is recognized as a nutritionally rich superfruit with medicinal and industrial importance. However, its inherently indeterminate growth habit causes asynchronous flowering and uneven fruit ripening, resulting in harvesting inefficiency with high labour costs and low fruit yield. To elucidate the molecular basis of shoot determinacy and early flowering, comparative transcriptome analysis of indeterminate and determinate shoot apices in an F_1_ hybrid population of *Lycium barbarum* identified 15 candidate genes associated with shoot growth termination and early flowering, including an *SP‐like* gene (*LbSP1*, *Lba0102749*) and a goji homologue of *SlSP5G* (*LbSP5G1*, *Lba0501086*) from the phosphatidylethanolamine‐binding protein (PEBP) family. CRISPR/Cas9‐mediated knockout of *LbSP1* and *LbSP5G1* in black goji berry produced homozygous single (CRLbSP1, CRLbSP5G1) and double determinate (2gCR *LbSP1* + *LbSP5G1*) lines exhibiting compact shoot architecture, reduced day‐length sensitivity, terminal flowering, and improved fruit yield. The double determinate line showed a significantly reduced number of leaves to the first inflorescence (7.25) compared to wild‐type (WT) (18.6), with fruit yield per plant of 174.94 g, followed by CRLbSP5G1 (64.55 g), CRLbSP1 (55.17 g), and WT (22.09 g). Transcriptomic and co‐expression analysis revealed extensive reprogramming of flowering regulatory networks, with *LbSP1* and *LbSP5G1* functioning as central regulatory hubs. Protein–protein interaction assays further established functional divergence between the two genes within a PEBP‐centred network governing meristem fate. These findings provide the first functional characterization of terminal flowering genes in goji, establishing a molecular framework for breeding compact, early‐flowering, and high‐yield cultivars optimized for mechanized harvesting.

## 1 Introduction

Goji berries ( *Lycium barbarum* L.) are deciduous woody perennials of the Solanaceae family that thrive in warm subtropical climates and are highly valued for their medicinal and nutritional properties (Amagase and Farnsworth 2011). Despite increasing demand, harvesting remains a major bottleneck limiting industry expansion. In Ningxia, the harvest period extends from June to October, requiring weekly fruit collection due to asynchronous ripening—a labour‐intensive process that conflicts with other cropping systems and incurs high costs. The indeterminate growth habit of goji leads to the coexistence of flowers and fruits at different developmental stages on the same plant, further complicating mechanized harvesting (Gong et al. 2022). A promising strategy to overcome these limitations is the development of determinate cultivars characterized by terminal flowering, synchronized fruit maturation, and compact architecture—traits that have enabled efficient mechanized harvesting in several crops (Kwon et al. 2020). Recently, germplasms exhibiting terminal flowering and consistent fruit maturation have been identified in *L. barbarum* , providing a foundation for mapping loci and identifying candidate genes controlling shoot architecture‐related traits (Gong et al. 2022). However, the molecular basis of growth habit regulation in goji berry remains comparatively underexplored, highlighting the need for functional genomic approaches to identify and characterize the key regulatory gene governing shoot determinacy in this crop.

At the molecular level, the *Centroradialis* (*CEN*)‐*TERMINAL FLOWER1* (*TFL1*)‐*Self‐Pruning* (*SP*) gene family, collectively referred to as the CETS family, plays a central role in controlling meristem identity and flowering patterns across plant species (Pnueli et al. 2001; McGarry and Ayre 2012). In *Antirrhinum majus* , *CEN* suppresses terminal flowering and its mutations lead to determinate growth (Bradley et al. 1996). *TFL1* in *Arabidopsis thaliana* delays floral transition, with mutants forming flowers prematurely (Bradley et al. 1997). Similar functional conservation has been observed in cereals, including rice (Nakagawa et al. 2002; Zhang et al. 2005) and barley (Comadran et al. 2012), and in grapevine, where disruption of *VvTFL1A* alters cluster size and branching (Fernandez et al. 2009). In tomato, the *SP* gene functions analogously, with recessive *sp* mutants exhibiting terminal flowering, compressed branching, and compact plant architecture, traits exploited to breed determinate varieties suitable for mechanized harvesting (Pnueli et al. 1998). Other CETS members act antagonistically to *TFL1/SP*: *SINGLE FLOWER TRUSS* (*SFT*) in tomato and *FLOWERING LOCUS T* (*FT*) in *Arabidopsis* promote floral transition and determinate growth (Liu et al. 2016), highlighting the importance of the relative expression balance between *FT‐like* and *TFL1/SP‐like* genes in controlling the pace and pattern of plant development.

Beyond the CETS family, additional transcription factors (TFs) modulate flowering and meristem maintenance. In *Arabidopsis*, *APETALA2* (*AP2*) modulates floral transition by repressing key floral integrators including *SOC1*, *AP1*, *FUL*, *SEP3*, and *AG*, while indirectly maintaining indeterminate growth via *TFL1* upregulation (Würschum et al. 2005; Yant et al. 2010; Krogan et al. 2012). Interactions between *AG* and *WUSCHEL* (*WUS*) further regulate stem cell maintenance and floral determinacy (Lenhard et al. 2001). In barley, *HvAP2* prevents premature spikelet initiation, and its mutation alters inflorescence architecture (Zhong et al. 2021). Other regulators, including MADS‐box genes (*SVP*, *SOC1*, *AGL24*), SQUAMOSA promoter binding protein‐like (SPL) TFs, including SPL1/2, and the SPL/miR156 module, fine‐tune flowering time and inflorescence development (Lee et al. 2000, 2007; Yu et al. 2002; Michaels et al. 2003; Klein et al. 1996; Preston and Hileman 2010). MYB TFs also participate in flowering control; EARLY FLOWERING MYB (EFM) and MYB106 modulate flowering through FT‐dependent pathways as dose‐sensitive repressors (Yan et al. 2014; Hong et al. 2021). Allelic variation in flowering regulators such as *BnaFTA2* and *BnaFTC6* further illustrates how natural diversity at these loci can be harnessed to control flowering time and seasonal adaptation in crops (Wan et al. 2025). Despite this extensive knowledge of the regulatory networks controlling indeterminate versus determinate growth in model systems, the functional roles of these regulators in goji berry remain largely unexplored.

Advances in genomics and genome editing now provide powerful tools to dissect these regulatory pathways in non‐model crops (Cao et al. 2021; Rehman et al. 2022). High‐quality genome assemblies of goji have facilitated genome‐wide identification of candidate genes (Cao et al. 2021; Xu et al. 2025), while RNA sequencing (RNA‐seq) enables systematic discovery of genes associated with flowering and developmental transitions (Chen et al. 2016). This integrative transcriptome‐guided strategy has been successfully applied across multiple crops including soybean, wheat, watermelon, Chinese cabbage, foxtail millet, and oilseed rape (Trick et al. 2012; Gu et al. 2017; Song et al. 2017; Gao et al. 2022; Tang et al. 2023; Zhan et al. 2023). CRISPR/Cas9‐mediated genome editing has further emerged as a robust platform for functional validation and targeted crop improvement—demonstrated across model Solanaceous crops (Lemmon et al. 2018; Kwon et al. 2020), and extended to medicinal and non‐model species including *Dendrobium officinale* (Li et al. 2024) and goji berry itself (Wang et al. 2021; Ai et al. 2023). The successful application of CRISPR/Cas9 in goji establishes the technical foundation necessary for functional characterization of shoot architecture regulators in this crop.

In this study, we aimed to accelerate the molecular breeding of goji berries through transcriptome‐guided functional genomics and genome editing. We performed the first comparative transcriptome analysis of the terminal shoot apices of indeterminate and determinate plants within an F_1_ hybrid population of *L. barbarum* to identify key regulators of flowering and growth determinacy, revealing 15 candidate genes associated with these processes. Among them, an *SP‐like* gene (*Lba0102749*) and a goji homologue of *SlSP5G* (*Lba0501086*)—identified and cloned from *L. barbarum* and designated *LbSP1* and *LbSP5G1*, respectively, were selected for CRISPR/Cas9‐mediated functional characterization. As stable transformation of *L. barbarum* has not yet been established, functional validation was performed in the closely related species *Lycium ruthenicum* (black goji berry; BGB), generating determinate and double‐determinate lines with compact architecture, terminal flowering, and improved yield potential. These findings provide a molecular framework for breeding compact, early‐flowering goji cultivars optimized for high‐density cultivation and mechanized harvesting, marking a key step toward modernizing goji crop improvement.

## 2 Material and Methods

### 2.1 Planting Population and Materials Selection

An intraspecific F_1_ hybrid population was generated by crossing *L. barbarum* cv. JiamengQi1 as the female parent with *L. barbarum* cv. Zhongkelüchuan1 (ZKLC1) as the male parent, and the resulting plants were planted in the field at the Northwest China Bio‐agricultural Center (38°28′05″ N, 106°16′23″ E, Yinchuan, Ningxia). The base population included more than 400 individual plants, among which several plants displaying contrasting terminal and non‐terminal shoot apex flowering characteristics were identified through three consecutive years of field observation (2020–2022). Before the onset of flowering, we selected three different types of shoot apex samples: N (non‐terminal shoot apices of indeterminate individual plants) (Figure S1A1–A3), TB (terminal shoot apices of determinate individual plants before apical termination) (Figure S1B1–B3), and TA (terminal shoot apices of determinate individual plants after apical inflorescence initiation at the apical meristem) (Figure S1C1–C3). All three shoot apex samples were collected in May for scanning electron microscopy (SEM) and in August for transcriptome sequencing and RT‐qPCR, corresponding to the active vegetative and peak flowering developmental windows respectively. For SEM analysis, shoot apices of approximately 2 mm in size were carefully excised from three individual plants per sample type. For transcriptome and RT‐qPCR, shoot apex samples from three individual plants per group, serving as three independent biological replicates, were immediately frozen in liquid nitrogen and preserved at −80°C until RNA extraction (Figure S1D,E).

### 2.2 Scanning Electron Microscopy

Shoot apex developmental patterns were comparatively examined across three sample types—N, TB and TA—as described in Section 2.1. For each sample type, a minimum of five shoot apices were processed for SEM analysis. Prior to fixation, bracts and visible floral organs were carefully removed under a dissecting microscope. Shoot apices were fixed overnight at 4°C in a solution of 2.5% glutaraldehyde and 2% paraformaldehyde in 0.1 M sodium phosphate buffer (pH 7.2), then washed three times in the same buffer (30 min per wash) and dehydrated through a graded ethanol series (30%, 50%, 70%, 80%, 90%, and three changes of 100%). Samples were dried using a Leica EM CPD300 critical point dryer, mounted on aluminium stubs, and sputter‐coated with gold to approximately 8‐nm thickness using a Leica EM ACE600 sputter coater. Coated samples were examined and imaged using an SU8100 field emission SEM (Hitachi, Japan) at an accelerating voltage of 5 kV. For gross morphological observations of intact shoot architecture, including sympodial branching patterns and terminal shoot formation, additional shoot samples were photographed using a Leica DVM6 digital light microscope.

### 2.3 RNA Extraction and Transcriptomic Analysis

Total RNA was extracted from shoot apex tissues representing three developmental stages—N, TA, and TB—with three independent biological replicates per group, and from shoot apical meristem (SAM) and immature fruit (S1 stage) tissues of wild‐type (WT) and gene‐edited lines (CRLbSP1, CRLbSP5G1 and 2gCR), with two independent biological replicates per genotype per tissue type. RNA quantity and purity were assessed using a NanoDrop 2000 spectrophotometer (Thermo Fisher Scientific, USA). High‐quality RNA samples were used for cDNA library preparation following the protocol described by Chen et al. (2017) and Zhan et al. (2023). Library quality was verified using an Agilent 2100 Bioanalyzer system prior to sequencing. Sequencing was performed on the Illumina Hi‐Seq2000 platform (Genosec Company, Wuhan, China) to generate 150‐bp paired‐end reads. Raw Illumina reads were processed with fastp (Chen et al. 2018) for adapter trimming and quality filtering using a 4‐bp sliding window approach, with reads shorter than 50 bp removed. Clean reads were aligned to *in‐house* data and the *L. barbarum* reference genome (1.68 Gb; Cao et al. 2021) using HISAT2 v2.1.0 (Kim et al. 2019). Read positions were annotated using ANNOVAR (Wang et al. 2010), with the majority of reads mapping to exon regions. Transcript assembly was performed using StringTie v1.3.6, and assemblies from all samples were integrated into a comprehensive transcript set using StringTie Merge (Pertea et al. 2015). Gene expression was quantified as transcripts per million (TPM) using RSEM v1.2.31 (Li and Dewey 2011), based on gene length and mapped read counts. The distribution of TPM values across samples was visualized using box plots (Li et al. 2010). Pearson correlation coefficients among samples were calculated from TPM values using the cor function in R v4.1.0, and correlation matrices were visualized using the ggplot2 package (https://cran.r‐project.org/web/packages/ggplot2/). Principal component analysis (PCA) was conducted using the *prcomp* function in R v4.1.0. Sequencing quality metrics and mapping statistics for all samples are provided in Tables S1 and S2.

### 2.4 Differential Expression and Functional Enrichment Analysis

Differentially expressed genes (DEGs) were identified across three pairwise comparisons (N vs. TA, N vs. TB, and TA vs. TB), each comprising three independent biological replicates per group, using the DESeq2 R package v1.10.1 (Love et al. 2014), which employs a model based on the negative binomial distribution. Raw read counts were used as input for DESeq2 differential expression analysis. Raw *p*‐values were adjusted for multiple testing using the Benjamini–Hochberg (BH) procedure to control the false discovery rate (FDR). Genes with an absolute log_2_ fold change (|log_2_FC|) ≥ 1 and an adjusted *p* ≤ 0.05 were defined as significantly differentially expressed. Overlapping DEGs across the three pairwise comparisons were identified and visualized using Venn diagram analysis. For visualization and clustering of expression patterns, transcript abundance was independently quantified as transcripts per million (TPM) using StringTie. TPM values of DEGs were log_2_‐transformed and subjected to hierarchical clustering analysis. Heatmaps were generated using the pheatmap R package v1.0.12 implemented in R v4.1.0 (http://www.r‐project.org/). Gene Ontology (GO) (Ashburner et al. 2000) and Kyoto Encyclopedia of Genes and Genomes (KEGG) (Kanehisa 2000) enrichment analyses were conducted to identify functional categories and biological pathways significantly associated with upregulated and downregulated DEGs in each comparison group. Enrichment analysis was performed using a hypergeometric test implemented in R (function *phyper*), and resulting *p*‐values were adjusted for multiple testing using the BH procedure. GO terms and KEGG pathways with adjusted *Q*‐values ≤ 0.05 were considered significantly enriched (Robinson et al. 2009).

### 2.5 Multiple Sequence Alignment and Phylogenetic Analysis

Amino acid sequences of *SP* and *SP5G* family members from tomato ( *Solanum lycopersicum* ) and *Arabidopsis thaliana* were obtained from the Phytozome v12.1 database (https://phytozome.jgi.doe.gov/pz/portal.html). Potential *L. barbarum* homologues were identified through BLASTP searches using tomato *SlSP* and *SlSP5G* amino acid sequences as queries against the *in‐house* datasets and *L. barbarum* genome database. Among the identified candidates, *Lba0102749* (*LbSP1*) and *Lba0501086* (*LbSP5G1*) exhibited the lowest E‐values, highest sequence similarity scores, and conserved phosphatidylethanolamine‐binding protein (PEBP) domain architecture consistent with tomato *SlSP* and *SlSP5G*, respectively. The functional annotation of *LbSP1* and *LbSP5G1* was further validated through BLASTP searches against the NCBI non‐redundant protein database (https://www.ncbi.nlm.nih.gov/protein). Multiple sequence alignments of *LbSP1* and *LbSP5G1* with their tomato and *A. thaliana* orthologs were performed using MUSCLE, and alignment visualization and editing were conducted using BioEdit v7.2.5. Aligned sequences were used to construct phylogenetic trees by the neighbour‐joining method with a Poisson correction model and pairwise deletion of gaps, implemented in MEGA v7, with 1000 bootstrap replicates to evaluate branch support. Phylogenetic analyses were performed using amino acid sequences.

### 2.6 Seed Germination, Callus Induction and Shoot Regeneration

Seeds of *L. ruthenicum* Murray were surface‐sterilized and germinated on Murashige and Skoog (MS) basal medium following the protocol described in our previous study (Ai et al. 2023). Leaf explants derived from 30‐day old seedlings were used for callus induction and subsequent shoot regeneration. All tissue culture procedures, including medium composition, plant growth regulator concentrations, and subculture intervals, were performed as previously described (Wang et al. 2021; Ai et al. 2023). Cultures were maintained at 25°C ± 2°C under a 16 h light/8 h dark photoperiod throughout all stages of callus induction and shoot regeneration. For each transformation event, a minimum of 30 explants were used to ensure sufficient statistical power for evaluating callus induction and regeneration frequencies.

### 2.7 CRISPR/Cas9 Constructs and Genetic Transformation

Single and multiplex CRISPR/Cas9 constructs for the targeted mutagenesis of *LbSP1* and *LbSP5G1* were generated following standard protocols (Brooks et al. 2014; Lemmon et al. 2018). Guide RNAs (gRNAs) were designed based on the *L. barbarum* coding sequences of *LbSP1* and *LbSP5G1* using the web‐based tool CRISPR‐GE (http://skl.scau.edu.cn/) (Xie et al. 2017), which ensured high specificity to minimize potential off‐target sites. Prior to gRNA design, the coding and protein sequences of *LbSP1* and *LbSP5G1* from *L. barbarum* were used as BLAST queries in BioEdit v7.2.5 to identify the corresponding orthologous loci in the *L. ruthenicum* genome. Genomic and coding sequence alignment of both genes using DNAMAN v8 confirmed conservation of the coding sequences, with complete identity at the gRNA target and PAM sequences between the two species, supporting the applicability of *L. barbarum* ‐derived gRNAs for CRISPR/Cas9‐mediated editing in *L. ruthenicum*. For single‐gene constructs, one pair of gRNAs (gRNA1–gRNA2), each 20 bp in length, was designed, whereas for multiplex constructs, two pairs of gRNAs (gRNA1–gRNA2 and gRNA3–gRNA4) were designed, corresponding to the target genes. gRNAs were chosen from coding regions (exons) with 40%–60% GC content and used as primer pairs (Table S3). Furthermore, potential off‐target sites for *LbSP1* and *LbSP5G1* gRNAs were predicted using Cas‐OFFinder (http://www.rgenome.net/cas‐offinder/) and CRISPR‐P v2.0 (http://crispr.hzau.edu.cn/CRISPR2/) with the canonical SpCas9 PAM (NGG) (Bae et al. 2014; Lei et al. 2014). Searches were performed against the *L. barbarum* genome, and *Solanum lycopersicum* reference genome owing to its high synteny and genetic similarity within the Solanaceae family. The parameters were set to allow for a maximum of three mismatches and no bulges (bulge size = 0). Specific gene fragments were amplified and verified by Sanger sequencing. Although genome‐wide off‐target analysis was not conducted in this study, stringent gRNA design criteria combined with PCR screening, TA cloning, and direct sequencing of target regions ensured that the observed edits corresponded to the intended loci with high confidence. Construct assembly was performed using the golden gate modular cloning method with slight modifications (Weber et al. 2011; Lemmon et al. 2018). In the first cloning step, gRNAs were inserted downstream of the *A. thaliana* U6 promoter into the acceptor vectors pICH47751 and pICH47761. Level‐1 modules, including pICH47732‐NOSpro::NPTII, pICH47742‐35S::Cas9, pICH47751‐AtU6pro::gRNA‐1, and pICH47761‐AtU6::gRNA‐2, were assembled into the binary level‐2 vector pAGM4723 following standard procedures (Lemmon et al. 2018). Consequently, two single CRISPR/Cas9 constructs targeting *LbSP1* and *LbSP5G1* (Figure S2A,B) and one multiplex construct targeting *LbSP1* + *LbSP5G1* simultaneously (Figure S2C) were generated. All primers used for construct assembly are listed in Table S3. The final binary vector pAGM4723 contained one (single) or two (multiplex) gRNA pairs driven by the *A. thaliana* U6 promoter, along with the gRNA scaffold, NOS terminator, 35S::Cas9, and NOSpro::NPTII cassette. These constructs were introduced into *Agrobacterium tumefaciens* strain EHA105 and subsequently used for the genetic transformation of BGB—selected as the transformation host given the current unavailability of an efficient transformation system for *L. barbarum* , following the detailed transformation protocol previously described (Ai et al. 2023) (Figure S2D). Transgenic plants were identified by PCR amplification using pAGM4723 vector‐specific primers (pAGM4723‐F/R) and target gene‐specific primers (Table S3).

### 2.8 Identification of Mutants and Validation

Genomic DNA was extracted from leaf tissues of T0 transgenic plants following the protocol described by Ai et al. (2023). PCR amplification using gene‐specific primers flanking the respective target sites was performed to confirm successful editing at *LbSP1* and *LbSP5G1* loci in single‐ and double‐edited lines. Sanger sequencing of PCR amplicons and chromatogram analysis were performed using SnapGene v6.1 (https://www.snapgene.com/) to determine the precise nature of induced mutations, including homozygous, biallelic, and heterozygous editing events at both target loci. To confirm transgene‐free status, PCR‐based detection was performed in homozygous gene‐edited lines using vector‐specific primers targeting the Cas9 nuclease and NPTII selectable marker sequences, with the CRISPR/Cas9 plasmid serving as a positive control and WT plants as a negative control. To assess the transcriptional consequences of *LbSP1* and *LbSP5G1* disruption, RNA sequencing was performed on SAM and immature goji fruit (S1 stage) tissues from WT and homozygous single‐ and double‐edited lines, with two biological replicates per genotype per tissue type. Total RNA extraction and library preparation were performed as described by Ai et al. (2023). Details of RNA‐seq data processing and differential expression analysis are provided in Section 2.3. To validate RNA seq results and confirm reduced expression of *LbSP1* and *LbSP5G1* in edited lines, RT‐qPCR was performed on three independent biological replicates per sample using MonAmp Chemo HS qPCR Mix (Monad, China; Code No. MQ00101). Gene expression levels were calculated using the 2^−ΔΔCt^ method, with *LbACTIN* serving as the internal reference gene (Zeng et al. 2014). RT‐qPCR was additionally performed on selected DEGs identified through RNA‐seq to provide independent expression validation. Detailed PCR conditions and primer design criteria have been described previously (Zeng et al. 2014; Ai et al. 2023). All primers used for mutant validation and RT‐qPCR are listed in Tables S3 and S4.

### 2.9 Co‐Expression Network Analysis

Weighted gene co‐expression network analysis (WGCNA) was employed to construct gene co‐expression modules and identify candidate genes associated with *LbSP1* and *LbSP5G1* knockout–mediated regulation of flowering and shoot architecture in *L. ruthenicum*. Transcriptome data from S1 fruit and SAM tissues of WT, single (*LbSP1* or *LbSP5G1*), and double (*LbSP1 + LbSP5G1*) gene‐edited lines were utilized as input for network construction. The overall analysis strategy followed our recent study (Ai et al. 2025). Briefly, genes with average expression levels below a minimum threshold (FPKM ≤ 1.0) were removed to eliminate noise from lowly expressed genes, and a subset of genes with a coefficient of variation greater than 0.5 was retained for network construction to focus on genes with meaningful expression variation across samples. Co‐expression network modules were generated using the WGCNA package in R (Langfelder and Horvath 2008), employing the automatic network construction function (blockwiseModules) with a Pearson correlation method and the following parameters: soft‐threshold power = 7, TOMtype = ‘unsigned’, mergeCutHeight = 0.25, deepSplit = 2, and minModuleSize = 30. Module‐trait correlations were calculated using the expression levels of *LbSP1* and *LbSP5G1* as trait variables to identify modules most strongly associated with the knockout phenotype. Hub genes within each module were defined based on module membership scores (kME ≥ 0.8) and gene significance values, prioritizing genes with the strongest within‐module connectivity.

### 2.10 Phenotypic Traits Data Collection and Statistical Analysis

Phenotypic assessment of homozygous single determinate and double determinate T0 mutant lines alongside WT plants was conducted under greenhouse conditions with a 16‐h light/8‐h dark photoperiod at 25°C ± 2°C. All transgenic and WT plants were grown under identical environmental conditions to ensure valid phenotypic comparisons. The following shoot architecture and yield‐related traits were measured: primary shoot height (cm), sympodial branch length (cm), number of sympodial branches, leaves to the first inflorescence, fruits per sympodial branch, fruits per five nodes, fruits per node, and fruit yield per plant (g). For each genotype, a minimum of five individual plants were evaluated per biological replicate, with the exact number of replicates indicated in the corresponding figure legends. Data are presented as mean ± standard deviation (SD). Statistically significant differences between transgenic lines and WT were determined using Student's *t*‐tests at a significance threshold of *p* < 0.05. All statistical analysis and graphical representation were performed using OriginPro v2024 (https://www.originlab.com/).

### 2.11 Yeast Two‐Hybrid (Y2H) and Luciferase Complementation Imaging (LCI) Assays

Protein–protein interactions were analysed using the yeast two‐hybrid assay (Y2H). The coding sequences of *LbSP1* and *LbSP5G1* were cloned into the GAL4 DNA‐binding domain (BD) to generate bait constructs, while the coding sequences of the nine candidate TFs (SPL, DELLA1, B3‐1/ARF1, MADS‐box, ERF, WUS, MYB, TCP and DOF1) were cloned into the activation domain (AD) vector to generate prey constructs. All recombinant plasmids were verified by sequencing prior to yeast transformation. The yeast strain AH109 was co‐transformed with the indicated bait and prey plasmids using the lithium acetate/polyethylene glycol method according to the manufacturer's instructions (Guo et al. 2023). Transformants were initially selected on double dropout medium (DDO; SD/−Leu/−Trp) to confirm successful co‐transformation and expression of both fusion constructs. Positive colonies were subsequently transferred onto quadruple dropout medium (QDO; SD/−Leu/−Trp/−His/−Ade) to assess strong protein–protein interactions under stringent selection conditions. Plates were incubated at 28°C for 3–5 days and colony growth was monitored as evidence of interaction (Ito et al. 2001; Guo et al. 2023). Empty BD vector co‐transformed with AD constructs was included as a negative control to exclude autoactivation, and reciprocal bait‐prey combinations were included to confirm interaction specificity.

Protein–protein interactions in planta were examined using the luciferase complementation imaging (LCI) assay in *Nicotiana benthamiana* leaves. The coding sequences of *LbSP1* and *LbSP5G1* were fused to the N‐terminal fragment of firefly luciferase (nLUC), while the coding sequences of the nine candidate TFs were fused to the C‐terminal fragment (cLUC) to generate the respective constructs (Chen et al. 2008). All recombinant plasmids were verified by sequencing and subsequently introduced into *Agrobacterium tumefaciens* strain GV3101 by electroporation. *Agrobacterium* cultures were grown overnight, collected by centrifugation, and resuspended in infiltration buffer (10 mM MgCl_2_, 10 mM MES, pH 5.6, and 100 μM acetosyringone) to an OD_600_ of approximately 0.5–0.8. Equal volumes of *Agrobacterium* suspensions carrying the nLUC and cLUC constructs were mixed and co‐infiltrated into fully expanded leaves of 4–6 week‐old *N. benthamiana* plants using a needleless syringe (Guo et al. 2025). Plants were maintained under normal growth conditions for 48 h post‐infiltration. Infiltrated leaves were subsequently sprayed with 1‐mM D‐luciferin sodium salt solution (Vazyme, DD1210‐02), and luminescence signals were detected using a NightSHADE LB 985 in vivo plant imaging system (Berthold technologies, Bad Wildbad, Germany). Reconstitution of luciferase activity resulting from protein–protein interaction was indicated by luminescence signal intensity (Chen et al. 2008; Guo et al. 2025). Empty vector combinations were included as negative controls to exclude non‐specific luminescence signals. All primers used for construct generation in Y2H and LUC assays are listed in Table S5.

## 3 Results

### 3.1 Shoot Apex Development

**FIGURE 1:**
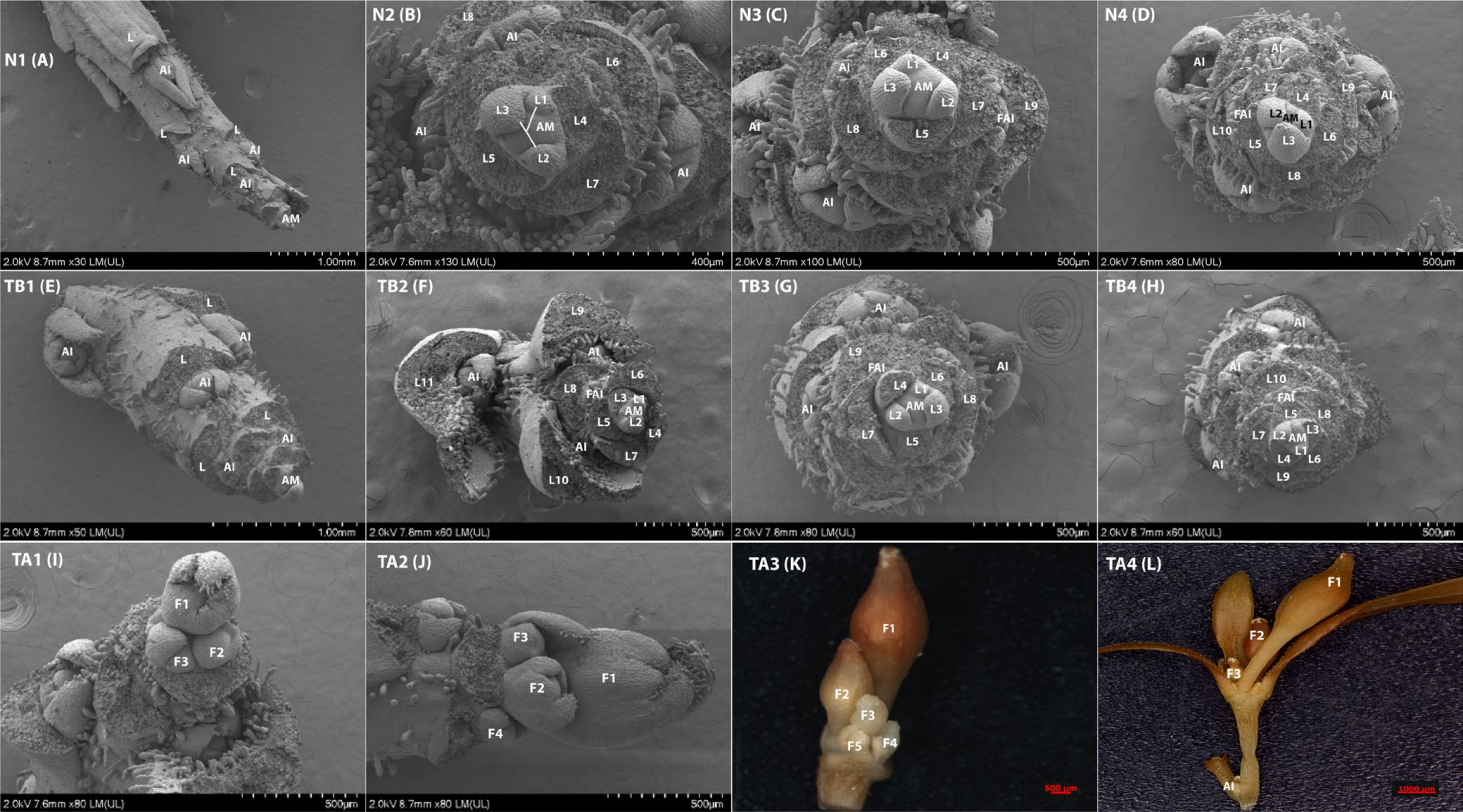
Scanning electron micrographs showing the developmental patterns of shoot apices in indeterminate and determinate *L. barbarum* plants. Leaf primordia were carefully dissected to expose axillary inflorescences and the apical meristem. (A–D) Non‐terminal shoot apices of indeterminate plants (N1–N4), showing continuous vegetative growth with axillary inflorescences arising after 8 (B), 9 (C), and 10 (D) leaves. (E–H) Terminal shoot apices of determinate plants before apical termination (TB1–TB4), showing shoot apex development comparable to indeterminate plants, with axillary inflorescence arising after 8 (F), 9 (G), and 10 (H) leaves, prior to apical cessation. (I, J) Terminal shoot apices of determinate plants after apical inflorescence initiation (TA1–TA2), showing early stage floral primordia development at the apical terminal inflorescence. (K, L) Terminal shoot apices (TA3–TA4) showing lateral flower development at the apical terminal inflorescence, with axillary inflorescences arising at each leaf node proximal to the apical meristem following shoot termination. AI, axillary inflorescence; AM, apical meristem; FAI, first axillary inflorescence; L1–L10, leaf number; F1–F5, flower number. Scale bars: 500 μm; 1000 μm.

In indeterminate shoots, the apex grows indefinitely and axillary inflorescences are produced in continuous succession along its flanks. Leaves exhibited an indeterminate spiral phyllotaxy pattern (Figure 1A), with the first and second leaf primordia forming a divergence angle of 137.5° around the apex—the divergence angle describing the relative angular positioning of two successively initiated organs as measured from the SAM centre. The second and third leaf primordia formed a divergence angle of 180° (Figure 1B), and divergence angles consistently changed between successive leaves. The apical meristem generated 8–10 leaves before the initiation of the first axillary inflorescence meristem, with the progressive enlargement of the apical dome giving rise to an inflorescence at the first apical meristem position (Figure 1B–D). The shoot apex retained vegetative growth identity throughout this process. In determinate shoots, apex growth prior to apical termination was morphologically comparable to that of indeterminate shoots (Figure 1E,F). However, the primary shoot apex underwent progressive size reduction in the determinate shoot, causing displacement of flower primordia from their normal positions and ultimately resulting in early shoot termination (Figure 1G,H). Shoot elongation ceased upon the formation of an apical inflorescence meristem at the apex (Figure 1I). The apical inflorescence meristems of determinate plants initiated significantly earlier than those of indeterminate plants, and determinate plants predominantly produced solitary flowers at the apex (Figure 1I). Following this vegetative to reproductive transition, axillary inflorescences arose at the axils of 8–10 leaves proximal to the apical meristem (Figure 1J–L). The initial apical dome of the reproductive meristem differentiated to produce the first flower of the inflorescence (Figure 1H), with each subsequent meristem emerging at a right angle and in an alternate direction relative to its predecessor (Figure 1I,J). The pedicel of the first flower may give rise to the second flower on either lateral side (Figure 1K,L). Notably, both the indeterminate and determinate materials used for morphological characterization and transcriptomic analyses were selected from within the same hybrid population of *L. barbarum* , comprising hundreds of individual plants derived from crosses between *L. barbarum* cultivars, as described in the Materials and Methods Section 2.1. As all sampled plants share the same hybrid genetic background and were grown under identical field conditions, the genetic diversity between the two groups is expected to reflect segregating variation within this single population rather than broad inter‐cultivar divergence. This intraspecific population design substantially minimizes confounding genetic background effects and supports the attribution of differentially expressed genes to developmental differences in shoot apex determinacy rather than to unrelated genetic divergence between the two growth types, thereby validating the comparative transcriptomic framework employed in this study.

### 3.2 Transcriptomic Analysis of Goji Shoot Apices

To investigate the molecular mechanisms underlying the distinct shoot apical developmental patterns observed between indeterminate and determinate goji plants (Section 3.1), transcriptome sequencing was performed on shoot apex samples from the same intraspecific population representing three developmentally distinct groups: indeterminate plants (N; Figure 1A–D) and determinate plants before apical termination (TB; Figure 1E–H) and determinate plants after apical inflorescence initiation (TA; Figure 1I–L). This three‐group sampling design was specifically chosen to capture the full developmental trajectory of shoot apex termination—from active indeterminate growth through the pre‐termination transition to post‐termination reproductive commitment, thereby enabling identification of genes associated with both early and terminal flowering regulation. As described in Section 2.1 and Figure S1, samples were collected from the apical meristematic region within approximately 2 mm of the shoot apex—the primary site of meristem identity and flowering transition gene expression during early developmental stages. RNA‐seq libraries generated high‐quality clean read pairs (Table S1). Approximately 90% of the reads were mapped to the *L. barbarum* reference genome, with 86% uniquely mapped, predominantly to exonic regions (Table S6). Gene expression profiling revealed clear distinctions among the nine samples—boxplot and correlation analyses confirmed high reproducibility across biological replicates, and PCA explained 52.7% of total variance, with samples clustering into three distinct groups corresponding to N, TB and TA, and tight grouping of replicates within each group (Figure S3). Differential expression analysis identified 33 614 expressed genes across all comparisons, of which 8154 were significantly differentially expressed, including 4119 in N versus TA, 2437 in N versus TB, and 1598 in TA versus TB (Figure S4). Volcano plots highlighted asymmetric DEG regulation across comparisons, with upregulated genes dominating in N versus TA, and N versus TB, whereas TA versus TB showed a greater proportion of downregulated DEGs (Figure S4A–C). Venn diagram analyses revealed 146 DEGs shared among all three comparisons, with the largest pairwise overlap between N versus TA and N versus TB (1554 genes), followed by N versus TA and TA versus TB (1014 genes) (Figure S4D,E).

GO enrichment analysis of DEGs revealed distinct functional categories associated with early and terminal flowering across all three comparisons, distributed across biological process (BP), molecular function (MF), and cellular component (CC) categories. In N versus TA, which exhibited the strongest overall enrichment, upregulated DEGs were predominantly associated with vacuole and lysosome components, DNA binding and kinase activity, while downregulated genes were enriched in DNA binding (Figure S5A,B). Similar patterns were observed in N versus TB, where upregulated genes were associated with vacuole, signal transduction, and lysosome functions and downregulated genes were enriched in plastid and signal transduction categories (Figure S6A,B). In TA versus TB, enrichment was comparatively less pronounced, with a small set of upregulated genes linked to RNA/DNA binding and organelle functions, and downregulated genes enriched in vacuole, lysosome, and DNA‐binding terms (Figure S6C,D). The strong enrichment of vacuole‐ and nucleus‐associated processes in N versus TA, including phosphatidylethanolamine‐binding protein (PEBP) family members such as *SP‐like* genes, suggests that these functional categories may play a key role in regulating flowering transitions in goji berry (Figure S5A; Table S7).

KEGG pathway analysis further supported these results. In N versus TA, upregulated genes were significantly enriched in phenylpropanoid biosynthesis, diterpenoid metabolism, starch and sucrose metabolism, cutin and wax biosynthesis, and plant hormone signalling, whereas downregulated genes were associated with protein processing in the endoplasmic reticulum and monoterpenoid biosynthesis (Figure S5C,D). In N versus TB, upregulated genes were enriched in phenylpropanoid and hormone signalling pathways, while downregulated genes were involved in endocytosis, spliceosome function, and protein processing in the endoplasmic reticulum (Figure S6E,F). In TA versus TB, upregulated genes were linked to diterpenoid, carotenoid, and zeatin biosynthesis, while downregulated genes were enriched in phenylpropanoid metabolism, cutin and wax biosynthesis, nitrogen metabolism, and starch and sucrose metabolism (Figure S6G,H). Together, GO and KEGG analyses indicate that pathways associated with secondary metabolism, hormone signalling, and cellular organelle function contribute to the regulation of early and terminal flowering in goji berry. Hierarchical clustering of 5143 DEGs based on TPM values revealed four distinct expression patterns across N, TA and TB shoot apices (Figure S7). Genes within each cluster exhibited coordinated expression, suggesting potential co‐regulation and involvement in related biological processes underlying flowering regulation (Figure S7A). Cluster 1 (2160 genes) showed consistently low expression across all groups, consistent with broad transcriptional repression in determinate tissues. Cluster 2 (2018 genes) was upregulated in TA and TB relative to N, suggesting activation during the transition toward reproductive development. Cluster 3 (805 genes) displayed broad upregulation in all sample types with stronger induction in TA and TB, indicating constitutive activation with enhanced expression during shoot termination. Cluster 4 (160 genes) comprised consistently highly expressed DEGs across all groups (Figure S7B), suggesting roles in fundamental developmental processes shared across growth types. These coordinated expression patterns support the existence of distinct transcriptional programs that collectively regulate the progression from indeterminate growth to early and terminal flowering in goji berries.

### 3.3 Identification of Terminal and Early Flowering Candidate Genes

**FIGURE 2:**
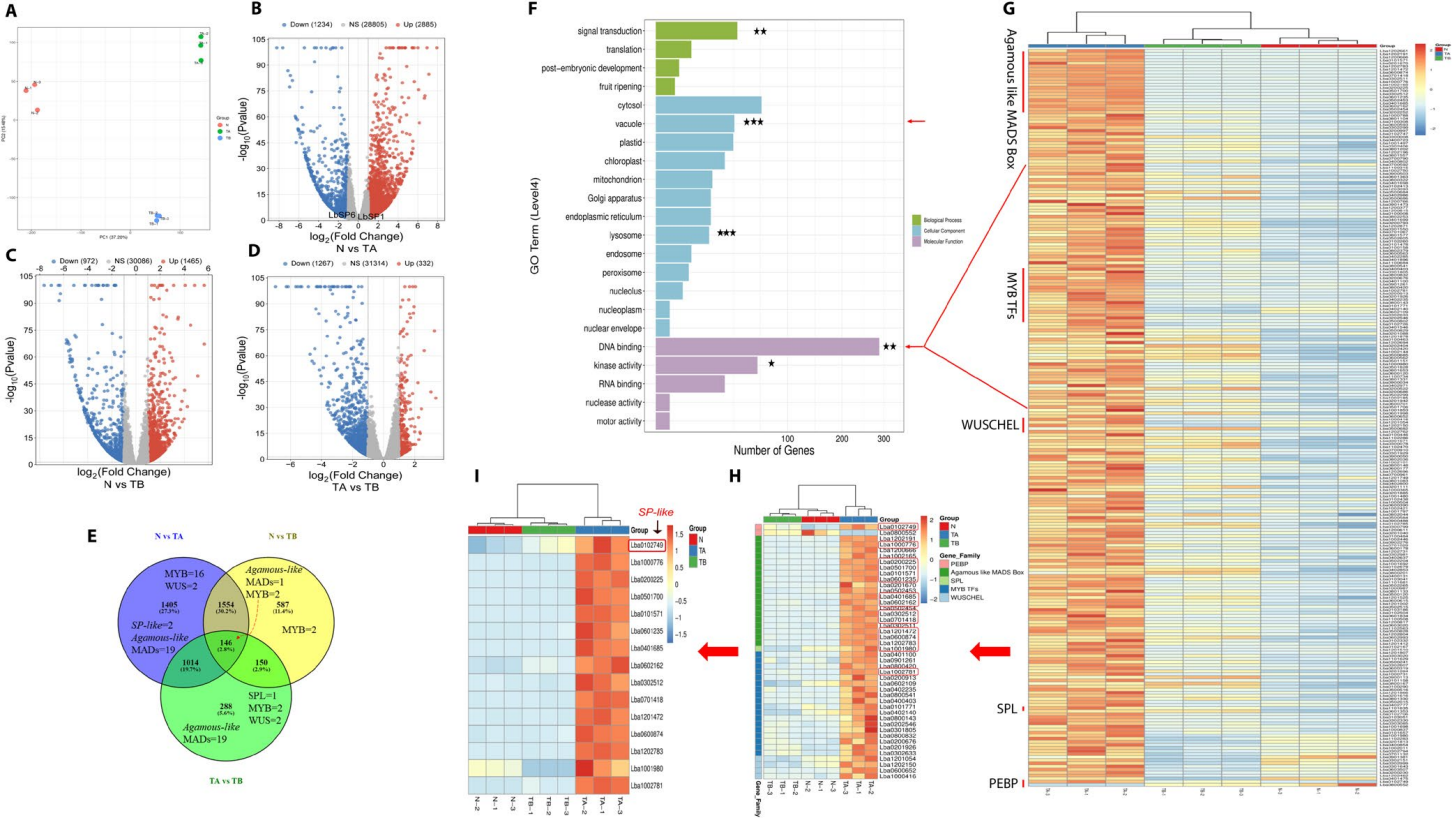
Transcriptome‐guided identification and progressive filtering of terminal and early flowering candidate genes from three shoot apex sample groups of *L. barbarum* . (A) Principal component analysis (PCA) of RNA‐seq data from three shoot apex groups, indeterminate (N1–N3), determinate before apical termination (TB1–TB3), and determinate after apical termination (TA1–TA3), with three independent biological replicates each, demonstrating clear genotype‐dependent sample separation. (B–D) Volcano plots of all differentially expressed genes (DEGs) in N versus TA (B), N versus TB (C), and TA versus TB (D), with log_2_FC values plotted against –log10 (FDR‐adjusted *p*‐values). Red and blue dots represent up‐ and down‐regulated DEGs (| log_2_FC | ≥ 1, FDR ≤ 0.05), and grey dots represent non‐significant genes. (E) Venn diagram representing the number of common and unique DEGs across the three pairwise comparisons. The N versus TA comparison contained candidate genes including two *SP‐like*, two *WUS*, 16 MYB, and 19 *Agamous‐like* MADS‐box; N versus TB contained two MYB genes; and TA versus TB contained 19 *Agamous‐like* MADS‐box, two *WUS*, two MYB, and one SPL gene. Three DEGs, one *Agamous‐like* MADS‐box, and two MYB TFs, were consistently shared across all three comparisons. (F) GO enrichment analysis of DEGs in all three comparisons, showing the top 30 enriched GO terms across biological process (BP), molecular function (MF), and cellular component (CC) categories (*p* ≤ 0.05). Asterisks indicate significantly enriched GO terms; red arrows indicate DEGs expression used for heatmap generation. (G) Heatmap of 241 DEGs enriched in DNA‐binding and vacuole‐related GO terms from the N versus TA comparison, showing expression patterns across nine shoot samples (N1‐N3, TA1‐TA3, TB1‐TB3). (H) Heatmap of 44 high‐confidence DEGs (log_2_FC ≥ 1, FDR ≤ 0.001) corresponding to terminal and early flowering regulatory gene families, including PEBP, *Agamous‐like* MADS‐box, SPL, MYB, and WUS. Red rectangle boxes indicate the 15 highly expressed DEGs in the terminal shoot apex group (TA1‐TA3) selected for further analysis. (I) Heatmap of 15 prioritized candidate genes, including one *SP‐like* gene (Lba0102749), 12 *Agamous‐like* MADS‐box (Lba1000776, Lba0200225, Lba0501700, Lba0101571, Lba0601235, Lba0401685, Lba0602162, Lba0302512, Lba0701418, Lba1201472, Lba0600874 and Lba1202783), one SPL gene (Lba1001980), and one MYB gene (Lba1002781). Red rectangle box and black downward arrow highlight *LbSP1* (Lba0102749) as the primary *SP‐like* candidate gene prioritized for CRISPR/Cas9‐mediated functional characterization based on its significant upregulation and phylogenetic conservation with tomato *SlSP*.

GO enrichment analysis of the 8154 DEGs across the three comparison groups was performed (Figure 2A–E), revealing significant enrichment of categories associated with flowering regulation. In the N versus TA comparison, which showed the strongest overall enrichment, DNA‐binding and vacuole‐related functional categories collectively comprised 241 significantly enriched DEGs (Figure 2F,G). From this dataset, 44 high‐confidence candidate genes (log_2_FC ≥ 1, FDR ≤ 0.001) showing elevated expression in terminal shoot apices were identified, belonging to key flowering regulatory families including two *SP‐like* genes (Lba0102749 and Lba0800552), four WUS genes, and multiple *Agamous‐like* MADS‐box genes (Figure 2H; Table S8). Only three DEGs—one *Agamous‐like* MADS‐box gene (Lba0602162) and two MYB TFs (Lba0201926, Lba0302633) were consistently shared across all comparisons (Figure 2E). Within the PEBP family, a total of 16 *SP‐like* gene copies were detected in the *L. barbarum* genome, of which only two (Lba0102749 and Lba0800552) were significantly upregulated in N vs. TA and clustered within expression subgroups associated with terminal flowering (Figure 2B,E–H; Table S8). Additional candidate flowering regulators included 19 *Agamous‐like* MADS‐box genes predominantly upregulated in N versus TA but downregulated in TA versus TB, one SPL gene (Lba1001980) significantly downregulated in TA versus TB, 18 MYB genes largely upregulated in N versus TA, and four WUS genes exhibiting a similar up–down expression trend across comparisons (Figure 2E–H; Table S8).

Further narrowing down these 44 candidate genes, 15 representative genes, including one *SP‐like* gene (Lba0102749), 12 *Agamous‐like* MADS‐box genes, one SPL gene, and one flower‐specific MYB TF—were selected based on their elevated expression in terminal shoot apices (TA) relative to indeterminate shoot apices (N and TB) (Figure 2I). It is hypothesized that these genes, particularly *SP‐like*, are likely involved in regulating determinate growth, compact plant architecture, and early flowering in goji. To validate the transcriptome results, all 15 genes were subjected to RT‐qPCR analysis (Figure 2I). Expression patterns were highly consistent with RNA‐seq data, with strong positive correlations observed across all pairwise comparisons (*R* ^2^ = 0.59–0.83, *p* < 0.05; Figures S8 and S9), confirming the reliability and robustness of the transcriptomic dataset.

Although the aforementioned candidate gene families, including *Agamous‐like* MADS‐box, WUS, SPL, and MYB family members, exhibited significant differential expression between determinate and indeterminate shoot apices, their established biological roles argue against direct involvement in shoot determinacy regulation. *Agamous‐like* MADS‐box genes are primarily associated with floral organ identity specification through the ABC model of flower development, functioning downstream of shoot determinacy signals rather than as initiators of determinate growth (Smyth et al. 1990; Bowman et al. 1991). Furthermore, the predominantly downregulated expression of *Agamous‐like* MADS‐box genes in TA versus TB, as the apex transitions toward reproductive commitment, is inconsistent with an upstream role in initiating shoot determinacy, further supporting their exclusion as primary targets. WUS genes, while critical for stem cell homeostasis, function in maintaining an undifferentiated meristem cell pool, a role fundamentally distinct from the termination of meristem activity that characterizes shoot determinacy (Mayer et al. 1998). SPL genes primarily mediate the age‐dependent pathway of flowering transition through gibberellin signalling, acting upstream of floral integrators rather than in shoot architecture determination per se (Klein et al. 1996; Gandikota et al. 2007). MYB TFs, despite their broad roles in plant development, lack direct functional precedent linking them to sympodial shoot termination or compact architecture in Solanaceae species (Dubos et al. 2010). In contrast, *SP/SP5G* homologues have been functionally established as central upstream regulators of shoot determinacy, flowering transition, and plant architecture across model and solanaceous crops (Pnueli et al. 1998; Soyk, Müller, et al. 2017; Soyk, Lemmon, et al. 2017; Lemmon et al. 2018; Kwon et al. 2020). Within the PEBP family, while Lba0800552 showed significant upregulation in N versus TA, phylogenetic analysis revealed that it clustered in a separate clade distant from the well characterized tomato *SlSP*, indicating weaker evolutionary conservation with functionally established *SP* orthologs associated with shoot determinacy regulation. In contrast, *LbSP1* (Lba0102749), clustered closely with *SlSP* gene within the SP/TFL1 subfamily, indicating strong phylogenetic support for its functional relevance to shoot determinacy (Figure S10). Additionally, *LbSP5G1* (Lba0501086), a goji homologue of *SlSP5G* identified through genome‐wide search of the *L. barbarum* genome, was incorporated as a complementary target based on its phylogenetic proximity to *SlSP5G* and the well‐established functional complementarity of the *SP/SP5G* regulatory module in controlling shoot determinacy in Solanaceous species. The selection of *LbSP5G1* alongside *LbSP1* was further motivated by the opportunity to simultaneously characterize the functional interaction between an *SP*‐type determinacy gene and its *SP5G*‐type florigen repressor counterpart, a combination that has proven highly informative in dissecting shoot architecture regulation in tomato and other Solanaceous species. Multiple sequence alignment (MSA) and phylogenetic analyses further confirmed that both proteins belong to the conserved SP/TFL1 subfamily within Solanaceae (Figure S10A,B). Therefore, based on the combined evidence from transcriptomic enrichment, expression specificity, RT‐qPCR validation, phylogenetic conservation, and previously established biological functions in related species, *LbSP1* and *LbSP5G1* were prioritized for CRISPR/Cas9‐mediated functional characterization. Whereas the remaining candidate gene families represent valuable targets for future functional investigation of flowering regulation and meristem development in goji berry.

### 3.4 Knockout of *LbSP1* and *LbSP5G1* via CRISPR/Cas9

**FIGURE 3:**
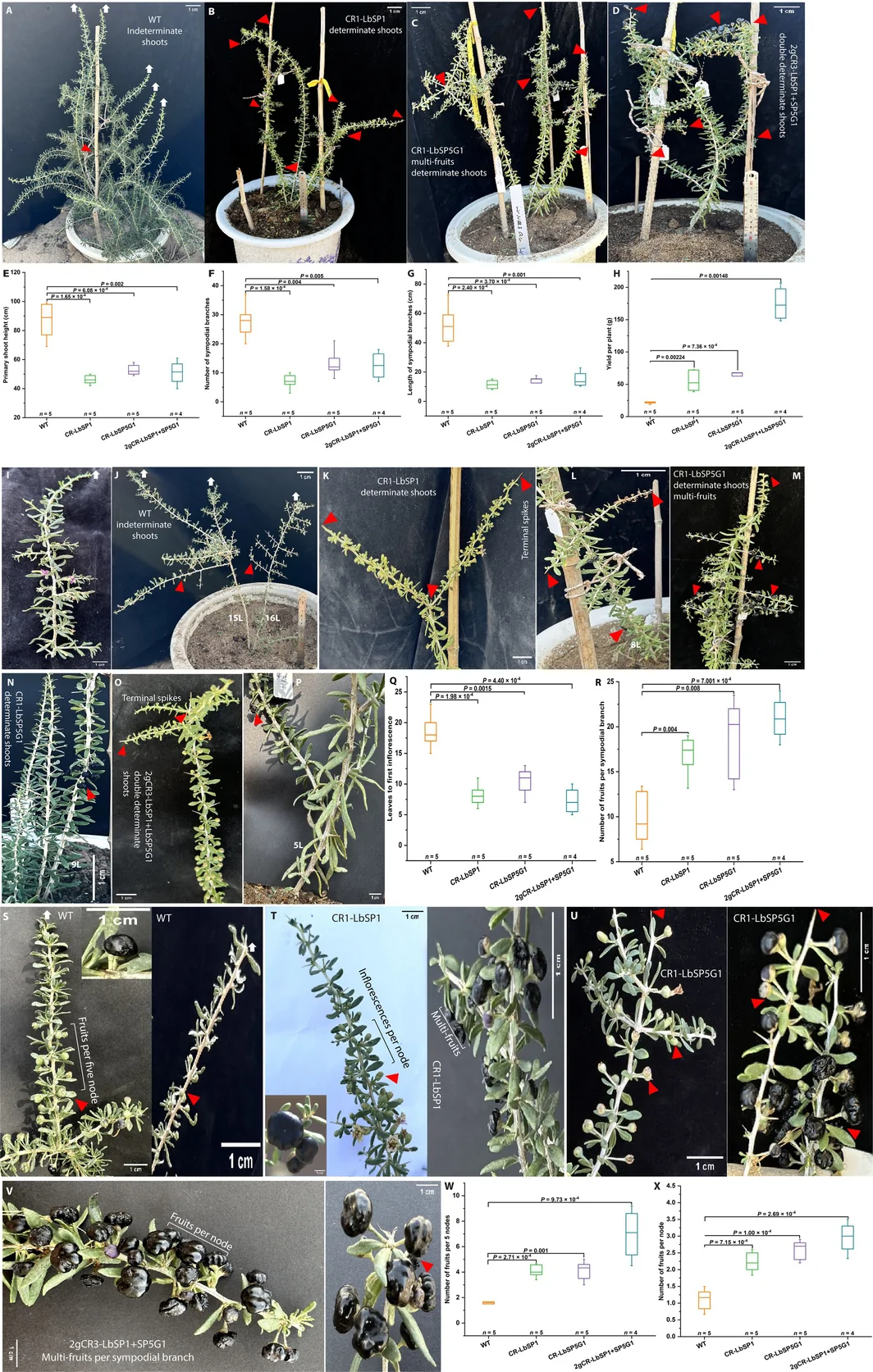
Phenotypic characterization of shoot architecture and fruit yield traits in WT and CRISPR/Cas9‐edited *L. ruthenicum* T0 homozygous single and double determinate lines. (A–D) Representative whole plant photographs comparing WT indeterminate (A), *LbSP1* single determinate (B), *LbSP5G1* single determinate (C), and *LbSP1* + *SP5G1* double determinate (D) T0 homozygous mutant lines, showing progressive compactness from WT to double determinate. (E–H) Quantification of shoot architecture and yield traits across all genotypes: primary shoot height—measured as the distance between the first leaf and the last inflorescence of the primary shoot (E), number of sympodial branches (F), length of sympodial branches (G), and fruit yield per plant (H). (I–P) Representative images of terminal branches showing leaf to inflorescence arrangement and sympodial branching patterns in WT indeterminate shoots (I, J), *LbSP1* single determinate shoots (K, L), *LbSP5G1* single determinate shoots (M, N), and *LbSP1* + *LbSP5G1* double determinate shoots (O, P). Numbers indicate leaf number within each sympodial cycle. (Q) Quantification of leaves to first inflorescence across all genotypes. (R) Number of fruits per sympodial branch. (S–V) Representative images of fruit‐bearing nodes showing fruiting patterns per five nodes in WT (S), *LbSP1* (T), *LbSP5G1* (U), and *LbSP1* + *LbSP5G1* double determinate (V) lines. (W) Number of fruits per five nodes. (X) Number of fruits per nodes. White arrowheads indicate indeterminate axillary inflorescence and continuous shoot growth. Red arrowheads indicate terminal spikes formation at the shoot apex, compact growth habit, and position of first inflorescence in determinate and double determinate shoots (I–P, S–V). L, leaf. Scale bars: 1 cm. Data are presented as mean ± SD. Box plots show the 25th–75th percentile range, with the centre line indicating the median and whiskers representing full data range. Statistical significance was assessed using a two‐tailed two‐sample student's *t*‐test. Sample sizes: *n* = 5 plants for WT and single determinate lines; *n* = 4 plants for double determinate lines.

To functionally validate the regulatory roles of *LbSP1* (Lba0102749) and *LbSP5G1* (Lba0501086) in shoot determinacy and flowering transition, CRISPR/Cas9‐mediated knockout was performed. Prior to CRISPR/Cas9 vector construction, the upstream promoter and coding sequences of *LbSP1* and *LbSP5G1* were examined to inform target site selection for genome editing. Given the functional conservation of the PEBP domain confirmed by MSA and phylogenetic analysis (Figure S10A,B), the conserved coding regions were selected as the primary targets for gRNAs design and CRISPR/Cas9 vector construction, ensuring effective disruption of gene function (Figure S2A–D). A multiplex CRISPR/Cas9 strategy carrying dual gRNAs targeting *LbSP1* and *LbSP5G1* was employed to simultaneously induce loss of function mutations in the two target genes and generate single (*LbSP1*, *LbSP5G1*) and double (*LbSP1* + *LbSP5G1*) edited lines (Figures 3 and S11). A total of 28 resistant seedlings carrying mutations in *LbSP1*, *LbSP5G1*, or both genes were successfully regenerated, including 12, 9, and 7 edited lines corresponding to gRNA1, gRNA2, and gRNA1/gRNA2 targets, respectively (Table S9). To validate successful genome editing, PCR amplification was performed using gene‐specific primers flanking the respective target sites in single‐ and double‐edited lines, yielding clear amplification of expected fragments, confirming successful targeting of both loci (Figure S11A). Sanger sequencing further resolved the precise nature of the induced mutations in the edited lines. In *LbSP1* single mutant and *LbSP1* + *LbSP5G1* double mutants, a 1‐bp insertion accompanied by a large 556‐bp deletion was detected at both targets (Figure S11B–D). For *LbSP5G1* single mutants, a 456‐bp deletion and an 8‐bp deletion were identified at the two target sites, while *LbSP1* + *LbSP5G1* double mutants exhibited a 1‐bp insertion and an 18‐bp deletion at the respective *LbSP1* and *LbSP5G1* target sites (Figure S11C–E). These mutations collectively resulted in frameshift mutations predicted to disrupt protein function in all edited BGB lines. Sanger sequencing chromatograms confirmed that these edited lines, including single mutants (CR1‐LbSP1, CR1‐LbSP5G1), and double mutants (2gCR3‐LbSP1 + LbSP5G1), carried homozygous mutations at each target locus in the T0 generation (Figure S11B–E).

Notably, the overall editing efficiency was remarkably high at 92.86%, with single mutant targets (*LbSP1*, *LbSP5G1*) achieving an efficiency of 67.86%, exceeding that of double mutants (*LbSP1* + *LbSP5G1*) by approximately 25% (Table S9). Of particular significance, homozygous edited plants were successfully recovered directly in the T0 generation—an outcome that is technically challenging in woody berry crops such as *L. ruthenicum*, which are inherently recalcitrant to transformation and regeneration. Among the T0 single‐ and double‐edited lines, homozygous editing efficiency accounted for 42.86%, whereas heterozygous mutations represented 57.14%, including 60% biallelic mutant lines (Table S9). This represents a notable achievement enabled by the optimized multiplexed CRISPR/Cas9 system and the refined tissue culture and regeneration conditions established in this study (Figure S2). The multiplexed system proved highly reproducible and efficient, generating reliable loss‐of‐function alleles carrying large deletions spanning essential regions of both target genes (Figure S11B–E). To confirm the transgene‐free status of the homozygous edited lines, PCR detection was performed using vector‐specific primers targeting the Cas9 nuclease and NPTII selectable marker sequences (Table S3). Amplification was observed only in the positive control containing the CRISPR/Cas9 plasmid, whereas no detectable bands were observed in either the WT or edited lines (Figure S11F). These results demonstrated that the homozygous single (*LbSP1*, *LbSP5G1*) and double (*LbSP1* + *LbSP5G1*) edited lines successfully retained the desired mutations while lacking detectable CRISPR/Cas9 vector integration, thereby confirming the generation of transgene‐free edited BGB plants.

To further validate functional disruption of *LbSP1* and *LbSP5G1*, RNA sequencing was performed on SAM and S1 fruit tissue samples from WT and transgenic single and double mutant lines. Among all PEBP family members, *LbSP1* and *LbSP5G1* expression levels were dramatically downregulated in single and double mutant lines relative to WT (Figure S12A–C), confirming effective gene knockout at the transcriptional level. RT‐qPCR analysis further corroborated these findings, demonstrating consistently reduced expression of both genes at goji fruit and shoot apices across all single and double homozygous mutant lines compared to WT (Figure S12D–F). Off‐target site analysis revealed that only one to three potential genomic sites were identified per guide sequence for *LbSP1*, all containing ≥ 2 mismatches with the intended target, while the *LbSP5G1* gRNA identified only two potential off‐target loci, each with three mismatches. No off‐target events with ≤ 2 mismatches were detected in either case (Table S10), confirming the high specificity of both gRNAs and the minimal likelihood of unintended Cas9‐mediated cleavage in goji.

Phenotypic assessment of the edited lines revealed that knockout of *LbSP1* and *LbSP5G1* effectively converted the sprawling indeterminate growth habit with inconsistent flowering into compact determinate growth with terminal flowering at all shoot apices, multiple flowers per node along sympodial branches, and consistent fruit yield compared to WT (Figure 3A–D,H–O). Distinct phenotypic effects were observed among the different mutant combinations. Specifically, *LbSP1* single mutant displayed *SP* determinate growth architecture characterized by compact condensed shoot and terminal spikes (Figure 3B,K), while the *LbSP5G1* single mutant exhibited *SP5G* determinate type with multi‐flowered inflorescences and increased fruit set per sympodial branch (Figure 3C,L,M). The double mutant (*LbSP1 + LbSP5G1*) combined both phenotypes, producing the most compact growth habit with the greatest degree of condensed shoots with terminal spikes, earliest flowering, and highest inflorescence density (Figure 3D,N,O). Interestingly, both determinate single mutants and double determinate mutant BGB plants exhibited compact shoot architecture with condensed growth and terminal spikes at the shoot apex (Figure 3B–D,K,M,O). All transgenic and WT plants were grown under the same environmental conditions to ensure valid phenotypic comparisons.

Quantitative assessment of shoot architecture traits confirmed significant differences among all genotypes. The *LbSP1* single mutant line produced more condensed shoots than the *LbSP5G1* mutant, with primary shoot height, number of sympodial branches, sympodial length, and yield per plant, averaging 46.2 cm, 7, 11.62 cm, and 55.17 g, respectively, compared to 53 cm, 13.40 cm, 13.78 cm, and 64.55 g in the *LbSP5G1* mutant (Figure 3A–H; Table S11). The loss‐of‐function *SP5G* allele additionally eliminated photoperiod sensitivity, producing faster flowering in both primary and axillary shoots with enhanced floral growth. Notably, all WT BGB plants produced more than 10 leaves before the first inflorescence (Figure 3J,Q), while this number was significantly reduced to approximately 5–6 leaves in double mutants (*LbSP1* + *LbSP5G1*) (Figure 3P,Q), compared to approximately 8–10 leaves in single mutant lines (Figure 3L,N,Q). In all other CRISPR‐edited lines, the maximum number of leaves produced before the first inflorescence was approximately 10–12. The double determinate mutant (*LbSP1* + *SP5G1*) also outperformed both single determinate mutants in all yield‐related parameters (Figure 3B–D), with primary shoot height, number of sympodial branches, and sympodial lengths, yield per plant averaging 51 cm, 12.75 cm, 16.75 cm, and 174.94 g, respectively (Figure 3E–H, Table S11). Furthermore, fruits per sympodial branch, fruits per five nodes, and fruits per node averaged 20.93, 6.98, and 2.96 for the double mutant, compared to 18.59, 4.086, and 2.58 for *LbSP1* single mutants, 16.77, 4.09, and 2.23 for *LbSP5G1* single mutants, and 9.86, 1.72, and 1.10 for WT, respectively (Figure 3I–P,R,S–X; Table S11). Collectively, these findings demonstrate that CRISPR/Cas9‐mediated disruption of *LbSP1* and *LbSP5G1* efficiently reprograms shoot determinacy, flowering behaviour, and fruit production in goji berry.

### 3.5 Transcriptomic Reprogramming and Regulatory Network Underlying *LbSP1* ‐ and *LbSP5G1* ‐Mediated Shoot Architecture Modification

To uncover the regulatory network governed by *LbSP1* and *LbSP5G1*, transcriptomes of *L. ruthenicum* S1 fruit and SAM from WT and gene‐edited lines were comparatively analysed. PCA confirmed clear genotype‐dependent separation of all samples, with PC1 and PC2 explaining 27.6% and 20.3% of total variance, respectively (Figure S13A). Differential expression analysis identified extensive transcriptional reprogramming across all comparisons: 3638 DEGs in WT‐S1 versus CRLbSP1‐S1, 14 196 DEGs in WT‐SAM versus CRLbSP1‐SAM, 12 510 DEGs in WT‐S1 versus CRLbSP5G1‐S1, 9073 DEGs in WT‐SAM versus CRLbSP5G1‐SAM, 2900 DEGs in WT‐S1 versus 2gCR‐S1, and 4832 DEGs in WT‐SAM versus 2gCR‐SAM (Figure S13B,C). Venn analysis revealed 3444 common DEGs across all pairwise comparisons (Figure S13D,E), which were subjected to GO and KEGG enrichment analysis. Enriched terms were predominantly associated with cell cycle regulation, meristem development, flower development, photosynthesis, and plant hormone signal transduction, with the double‐edited line exhibiting the most extensive transcriptomic reprogramming (Figure S13F,G; Tables S12 and S13). Hierarchical clustering of DEGs from enriched GO and KEGG terms revealed that key flowering regulators were broadly downregulated in gene‐edited lines relative to WT, with the most pronounced reduction observed in the double‐edited lines (Figure S14). Notably, key regulators of flowering transition and meristem identity, including MADS‐box (*Lru01G01302*, *Lru01G02315*, *Lru03G02465*, *Lru06G00884*, *Lru07G00816*, *Lru07G00817*, *Lru08G00280*), *MYB* TFs (*Lru01G02919*, *Lru08G00052*, *Lru08G00380*, *Lru08G02055*), *SEPALLATA1‐like* (*Lru12G02646*), *LEAFY/FLORICAULA* (*Lru03G02815*), *WUS* (*Lru10G00375*, *Lru12G01063*), *AP1‐like* (*Lru05G02444*), *SPL* (*Lru01G00466*), *PEBP‐FT* (*Lru01G01068*), *CONSTANS6* (*Lru05G01183*), *EMBRYONIC FLOWER* (*Lru04G01817*, *Lru04G01818*), and *EARLY FLOWERING4‐like* (*Lru09G00988*, *Lru09G00989*, *Lru09G00991*), showed consistently reduced expression in both single and double gene‐edited lines across SAM and S1 tissues (Figure S14A–C). These results support the conclusion that *LbSP1* and *LbSP5G1* function as key regulators of meristem fate determination, and their disruption triggers coordinated transcriptomic reprogramming that promotes a shift toward a more determinate and compact shoot architecture in goji berry.

To further characterize the gene regulatory networks mediated by *LbSP1* and *LbSP5G1*, WGCNA identified eight co‐expression modules from the DEG dataset (Figure S15A,B). Among these, the red module (143 genes; *R* = 0.95, *p* = 5.2e−12) showed the strongest positive correlation with *LbSP5G1*, with *LbSP5G1* itself emerging as a hub gene directly co‐expressed with WRKY, MYB, TCP, B3/AFRs, and AP2/ERF TFs, indicating a central role in coordinating flowering‐related transcriptional programs (Figure S15D). In the grey module (157 genes; *R* = 0.12, *p* = 0.57), *LbSP1* was co‐expressed with WUS and indirectly associated with ERF, NAC, and DOF TFs, suggesting its involvement in meristem identity regulation and flowering transition (Figure S15E). Full description of remaining modules and their associated gene networks are provided in supplementary figures (Figure S15). RT‐qPCR validation of 16 candidate genes representing key TFs families confirmed consistent downregulation in the SAM of all edited lines, in agreement with RNA‐seq results. A subset of genes, including MYB1‐MYB3, bHLH1, WUS1‐WUS2 and DELLA1, showed contrasting upregulation in S1 fruits (Figure S16), suggesting tissue‐specific regulatory effects associated with *LbSP1/LbSP5G1* disruption and highlighting the complexity of flowering network modulation in goji berry.

**FIGURE 4:**
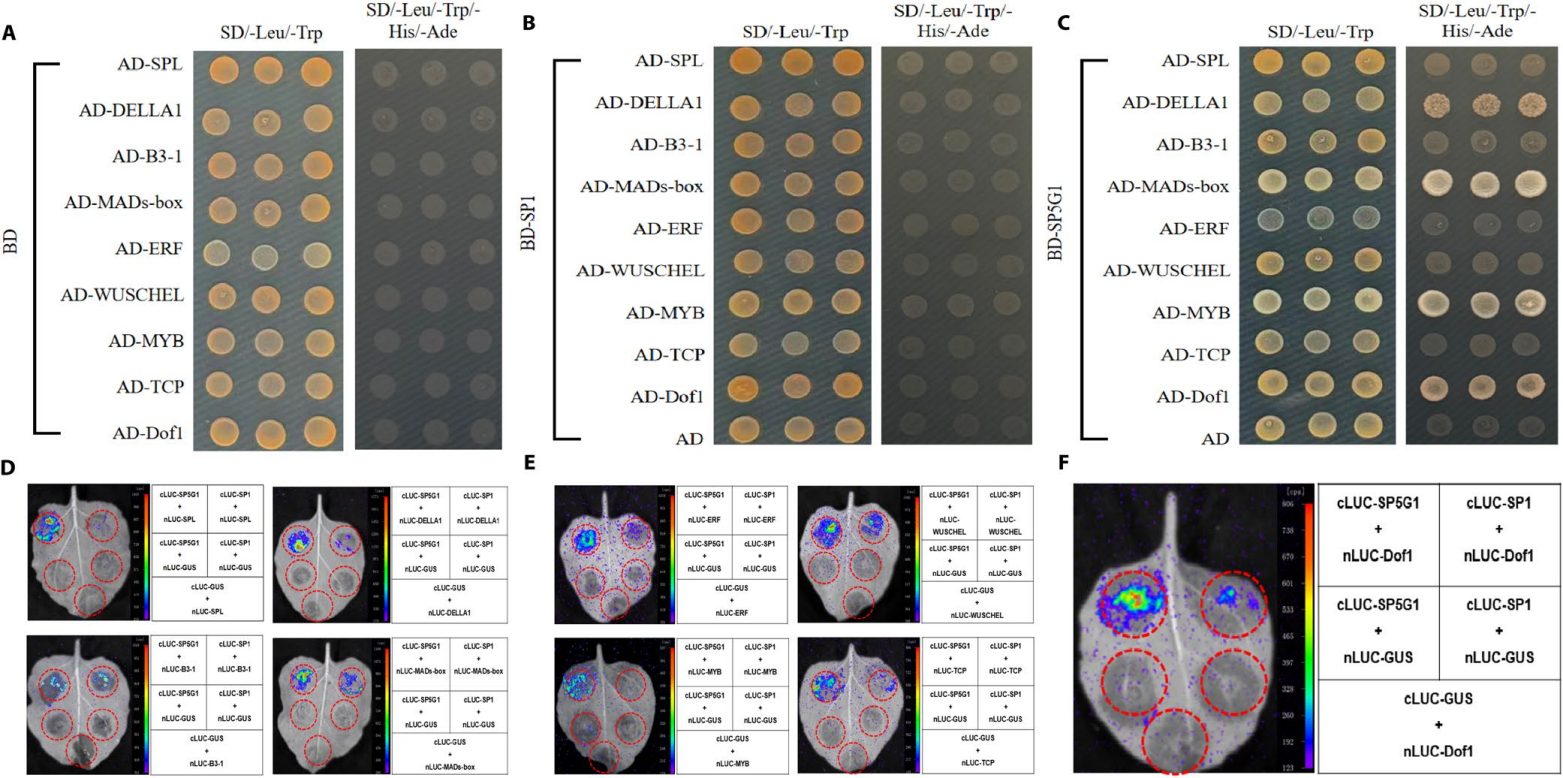
Differential protein–protein interaction profiles of *LbSP1* and *LbSP5G1* with nine flowering regulatory transcription factors (TFs)—SPL, DELLA1, B3‐1/ARF1, MADS‐box, ERF, WUSCHEL, MYB, TCP and DOF1, revealed by yeast two‐hybrid (Y2H) and luciferase complementation imaging (LCI) assays. (A–C) Y2H interaction assays. For each panel, left plates show growth on permissive double dropout medium (SD/‐Leu/‐Trp) confirming successful co‐transformation of BD and AD constructs; right plates show growth on stringent quadruple dropout medium (SD/‐Leu/‐Trp/‐His/‐Ade) indicating protein–protein interactions. Colony growth on QDO indicates a positive interaction. (A) Negative control assays using an empty BD vector co‐transformed with AD‐fused TF constructs, confirming the absence of autoactivation. (B) Interaction assays for BD‐*LbSP1* with AD‐fused TFs, showing no detectable growth on QDO medium. (C) Interaction assays for BD‐*LbSP5G1* with AD‐fused TFs, showing strong selective interactions with DELLA1, MADS‐box, MYB and DOF1 on QDO medium. (D–F) Representative luminescence images from LCI assays in *Nicotiana benthamiana* leaves showing reconstituted luciferase activity as an indicator of protein–protein interaction. Quantification of LCI luminescence signals for *LbSP5G1* combinations with all nine candidate TFs, showing strong signals with SPL, DELLA1, MADS‐box, WUS, MYB and DOF1, whereas quantification of LCI luminescence signals for *LbSP1* combinations shows moderate signals with DELLA1, B3‐1/ARFs, MADS‐box, ERFs, WUS, TCP and DOF1 (D–F). Empty vector combinations with GUS served as negative controls for non‐specific luminescence. Protein expression of nLUC and cLUC fusion constructs was confirmed by immunoblot analysis using anti‐luciferase and anti‐cLUC antibodies respectively.

To investigate protein–protein interactions within the *LbSP1*/*LbSP5G1* regulatory network, nine candidate TFs—SPL, DELLA1, B3‐1/ARF1, MADS‐box, ERF, WUS, MYB, TCP and DOF1 were selected based on transcriptomic and co‐expression analyses for Y2H and LCI assays. All co‐transformants grew normally on SD/‐Leu/‐Trp medium, confirming successful transformation and expression of fusion constructs. Importantly, co‐transformations of empty BD with AD constructs did not grow on SD/‐Leu/‐Trp/‐His/‐Ade, indicating the absence of autoactivation and validating the specificity of the system (Figure 4A). Y2H screening under stringent selection conditions revealed that *LbSP1* did not form stable binary interactions with any tested TF, whereas *LbSP5G1* showed strong selective interactions with DELLA1, MADS‐box, MYB, and DOF1 (Figure 4B,C). To validate these interactions in a plant cellular context, LCI assays were performed in *Nicotiana benthamiana* leaves. Consistent with the Y2H results, strong luminescence signals were detected for *LbSP5G1* in combination with SPL, DELLA, MADS‐box, WUS, MYB and DOF1 (Figure 4D–F). For *LbSP1*, moderate signals were detected with DELLA, B3‐1/ARFs, MADS‐box, ERFs, WUS, TCP and DOF1, suggesting indirect or context‐dependent interaction capacity that is detectable in planta but not under stringent yeast conditions (Figure 4D–F). Collectively, these results reveal a clear functional divergence between *LbSP1* and *LbSP5G1*: *LbSP5G1* acts as a direct interaction hub forming stable complexes with key flowering regulators, while *LbSP1* exhibits more selective and indirect interaction behaviour. Together with the transcriptomic and co‐expression data, these findings delineate a complex regulatory network involving MADS‐box, TCP, SPL, MYB, ERF, WUS, WRKY and AP2‐like TFs that coordinately regulate flowering and meristem development, and whose disruption by *LbSP1/LbSP5G1* knockout contributes to the modified shoot architecture observed in BGB.

## 4 Discussion

### 4.1 Shoot Determinacy and Its Agricultural Implications for Mechanized Goji Harvesting

The transition from vegetative to reproductive growth in flowering plants is governed by intricate regulatory networks that integrate environmental signals, including photoperiod and vernalization, with endogenous developmental cues through central integrator genes such as FLOWERING LOCUS T (*FT*) (Corbesier et al. 2007; Andrés and Coupland 2012; Song et al. 2013). Flowering initiation timing is a critical life‐history trait that directly determines reproductive success (Simpson and Dean 2002; Amasino 2010; Srikanth and Schmid 2011; Yan et al. 2014). Within this regulatory framework, members of the PEBP gene family, particularly TFL1, CEN and SP, play central roles in controlling shoot meristem identity and the transition between indeterminate and determinate growth. In *Arabidopsis*, TFL1 maintains inflorescence meristem identity and indeterminate growth, with *tfl1* mutants forming terminal flowers after rosette leaf production (Bradley et al. 1997), while CEN gene expression at the inflorescence apex marks the transition to terminal shoot growth (Bradley et al. 1996). *SP*, the tomato homologue of *CEN* and *TFL1*, controls sympodial shoot determinacy, with recessive *sp* mutants displaying compact, terminal‐flowering growth habits that have proven highly advantageous for mechanized harvesting (Pnueli et al. 1998). The functional conservation of *CEN*, *TFL1* and *SP* across higher plants in maintaining meristem vitality and controlling growth termination has been further demonstrated in ground cherries and other Solanaceous species (Li, Yang, et al. 2018; Lemmon et al. 2018; Zsögön et al. 2018; Kwon et al. 2020), establishing PEBP‐mediated determinacy as a broadly applicable target for crop architecture improvement.

The agronomic significance of shoot determinacy is particularly pronounced in berry crops such as goji berry, where the co‐occurrence of ripe fruits, immature fruits, and flowers on the same branch at the same time presents a fundamental challenge to mechanical harvesting (Chen et al. 2024). Currently, goji harvesting relies predominantly on manual labour, resulting in low picking efficiency and high production costs—a situation further exacerbated by the rapid migration of rural workers to urban areas and rising agricultural labour costs (Junfa et al. 2009; Chen et al. 2024). Developing compact, synchronized‐flowering goji cultivars suitable for mechanized harvesting (Li, Yang, et al. 2018; Guo et al. 2019) suggests that engineering shoot determinacy in goji could similarly improve harvest synchrony and mechanization compatibility. Flower production and growth termination at the branch apex promote uniform fruit ripening, providing both theoretical support and practical direction for breeding compact goji cultivars amenable to agricultural mechanization (Alvarez et al. 1992; Guo et al. 2019). In this context, the present study targeted *LbSP1* and *LbSP5G1* (PEBP family members) identified through comparative transcriptome analysis of contrasting growth habit types in *L. barbarum* for CRISPR/Cas9‐mediated functional characterization, with the goal of establishing the molecular basis for compact, determinate growth in goji berry and providing a genomic foundation for breeding cultivars compatible with mechanized harvesting operations.

### 4.2 Comparative Developmental Characterization of Goji Shoot Apices and the Conserved Role of PEBP Genes in Shoot Determinacy

The morphological distinction between indeterminate and determinate shoot apices in goji berry, characterized by continuous vegetative growth in indeterminate shoots versus meristem termination upon apical inflorescence initiation in determinate shoots (Figure 1)—mirrors the developmental transition controlled by *TFL1*/*SP‐like* PEBP genes in model and crop species. The regulatory role of *TFL1* and its homologues in controlling shoot determinacy is well established. In *Arabidopsis*, *TFL1* also suppresses premature floral fate at the apex as well as maintains inflorescence identity, with *tfl1* mutant switching from lateral floral meristems to terminal floral organs—a phenotype associated with reduced apical meristem size and disruption of the normal alternation between vegetative and reproductive growth (Smyth et al. 1990; Shannon and Meeks‐Wagner 1991; Bradley et al. 1997). Loss of *TFL‐like* function, together with a reduction in apical meristem size, promotes determinate inflorescence growth across multiple plant lineages including Brassicaceae and Scrophulariaceae, while gain‐of‐function mutations in *TFL‐like* genes promote indeterminacy (Alvarez et al. 1992). A directly comparable regulatory pattern occurs in tomato, where *SP*—the TFL1/CEN homologue—controls sympodial shoot determinacy. In determinate *sp* mutant plants, the number of vegetative nodes per sympodial units progressively decreases until consecutive inflorescences emerge, reflecting a disruption of the normal alternation between vegetative and reproductive phases without altering the overall sympodial framework (Yeager 1927; Macarthur 1932; Pnueli et al. 1998). This functional conservation of PEBP‐mediated determinacy has been further validated through CRISPR/Cas9‐mediated editing of *SP* orthologues in ground cherry and other Solanaceous species for crop architecture improvement (Li, Yang, et al. 2018; Lemmon et al. 2018; Zsögön et al. 2018; Kwon et al. 2020). The developmental patterns observed in goji shoot apices, including the progressive reduction in leaf number before inflorescence initiation in determinate plants—are strikingly consistent with these established *SP*‐mediated determinacy mechanisms, supporting the functional relevance of *LbSP1* and *LbSP5G1* in regulating shoot apex termination in *L. barbarum* .

Comparative transcriptome analysis of the three contrasting shoot apex groups identified 8154 DEGs (Figure S4), with functional enrichment revealing predominant associations with lysosome and vacuole functions, kinase activity, DNA‐binding, phenylpropanoid biosynthesis, carbohydrate metabolism, plant hormone signal transduction, and spliceosome‐related processes (Figures S5 and S6), consistent with previous reports on transcriptional regulation of shoot development in related species (Hua et al. 2016; Wang et al. 2016; Cai et al. 2020; Gao et al. 2021; Takase et al. 2022). Hierarchical clustering further revealed coordinated transcriptional sub‐clusters associated with meristem determinacy, with sub‐clusters I and II showing reduced expression and sub‐clusters III and IV exhibiting elevated expression (Figure S7A,B), consistent with earlier reports on meristem‐specific transcriptional organization (Huang et al. 2017; Li, Fang, et al. 2018). Further, identification of 44 key DEGs associated with terminal and early flowering regulatory families, including PEBP, *Agamous‐like* MADS‐box, SPL, MYB, and WUS, and the prioritization of 15 candidates based on elevated expression in determinate shoot apices implicate these gene families in shoot apex growth termination and early flowering in goji berry (Figure 2H,I). These transcriptomic findings are consistent with established regulatory frameworks across plant species. MADS‐domain TFs play a central role in floral transitions, *AGL20/SOC1* and *FUL* promote flowering, while *FLC*, *FLM/MAF1* and *SVP* act as repressors (Michaels et al. 2003), and overexpression of *SOC1* from *B. rapa* in *B. napus* induced early flowering through activation of *LFY* and *AP1* (Hong et al. 2012). *AGL24* is activated in the SAM during floral transition and its suppression delays flowering while overexpression accelerates the transition (Yu et al. 2002). MADS‐box genes (SEP1–SEP3) regulate floral organ identity (Huang et al. 1995), and in *Isatis indigotica*, silencing *IiSEP4* delayed flowering while its overexpression in *Arabidopsis* promoted early flowering (Ma et al. 2022; Pu et al. 2020). SPL TFs mediate signals from autonomous and photoperiodic pathways, activating FUL, AP1, and LFY, to determine floral meristem identity (Klein et al. 1996; Preston and Hileman 2010; Wellmer and Riechmann 2010). MYB TFs modulate flowering through diverse mechanisms—EFM MYB represses FT to prevent premature floral induction under unfavourable conditions (Yan et al. 2014), while MYB106 mutation causes early flowering and its overexpression delays the transition (Hong et al. 2021). Importantly, PEBP members including *SP* and *SP5G* modulate shoot architecture and flowering transitions across Solanaceous species (Soyk, Müller, et al. 2017; Lemmon et al. 2018; Kwon et al. 2020). While these gene families represent important components of the broader flowering regulatory network in goji, their differential expression patterns—characterized by upregulation in N versus TA but downregulation in TA versus TB for MADS‐box and WUS genes—suggest roles in downstream floral organ specification and meristem maintenance rather than primary shoot determinacy control, supporting the prioritization of *LbSP1* and *LbSP5G1* as the central upstream regulators targeted in this study.

Importantly, *SP5G* orthologues function as photoperiod‐dependent repressors of flowering in tomato. *SP5G* delays flowering under long‐day conditions but remains largely inactive during short days (Soyk, Müller, et al. 2017). This photoperiodic sensitivity has direct implications for the functional behaviour of *LbSP5G1* in goji berries. The loss of *LbSP5G1* function observed in our edited lines likely reduces day‐length sensitivity, promoting a more determinate flowering pattern and earlier, more synchronized fruit set, particularly under long‐day conditions characteristic of the goji production environment. However, under short‐day or cooler temperature regimes, premature determinacy could shorten the reproductive phase and potentially reduce total yield. These genotype × environment interactions suggest that fine‐tuning *LbSP1* and *LbSP5G1* function—through optimization of *SP* allelic variants or modulation of their cis‐regulatory activity—may be necessary to balance flowering duration and yield stability across production regions. Future studies evaluating the edited lines under contrasting photoperiod and temperature conditions will be essential to assess their environmental adaptability and to develop environment‐specific ideotypes suitable for mechanized harvesting and consistent fruit production across goji cultivation zones.

### 4.3 CRISPR/Cas9‐Mediated Shoot Architecture Modification in BGB: Functional Parallels With Solanaceous Crops and Implications for Cultivar Development

The present study demonstrates that CRISPR/Cas9‐mediated knockout of *LbSP1* and *LbSP5G1* effectively recapitulates and extends the architectural modifications previously achieved through *SP* and *SP5G* editing in tomato and ground cherry (Li, Yang, et al. 2018; Lemmon et al. 2018; Zsögön et al. 2018; Kwon et al. 2020), establishing goji berry as a new member of the Solanaceous crops amenable to PEBP‐mediated architecture improvement through genome editing. The broad utility of CRISPR/Cas9 for targeted agronomic improvement across Solanaceous species, including tomato (Brooks et al. 2014; Rodríguez‐Leal et al. 2017; Kwon et al. 2020), potato (Wang et al. 2015), *Physalis* (Lemmon et al. 2018), eggplants (Maioli et al. 2020), and BGB (Wang et al. 2021; Ai et al. 2023)—underscores the translational value of this approach. Notably, the complexity of the regulatory networks governing these traits does not preclude targeted manipulation, as exemplified by the finding that a regulatory module involving the circadian clock component *RhLHY* and the *trehalose‐6‐phosphate phosphatase RhTPPF* suppresses flowering under low light conditions through altered sugar allocation in rose (*Rosa hybrida*) (Fan et al. 2024)—demonstrating that precise editing of key regulatory nodes within complex networks can effectively reshape plant architecture.

The high editing efficiency and successful recovery of transgene‐free homozygous T0 lines in a single transformation event—a technically significant achievement for this recalcitrant woody berry crop—demonstrates the robustness of the multiplexed CRISPR/Cas9 platform established in this study and its applicability for rapid functional genomics in *Lycium* (Figure S11). The phenotypic outcomes in gene‐edited goji lines closely parallel those reported in tomato and ground cherry, with important species‐specific distinctions. Single *LbSP1* mutants displayed *SP*‐type compact determinate growth with terminal shoot condensation, while *LbSP5G1* single mutants exhibited *SP5G*‐type increased inflorescence density and accelerated flowering, consistent with the distinct but complementary roles of *SP* and *SP5G* orthologs in regulating sympodial shoot cycling and day‐length sensitivity, respectively (Soyk, Müller, et al. 2017; Kwon et al. 2020). Double determinate (*LbSP1* + *LbSP5G1*) lines produced the most compact architecture with a significant improvement in fruit yield per plant—mirroring the additive effects of *SP SP5G* double mutations in tomato and demonstrating the combined value of simultaneously disrupting both regulatory nodes (Soyk, Müller, et al. 2017; Kwon et al. 2020). The significant reduction in leaves before the first inflorescence in double mutants suggests accelerated floral transition and a shortened vegetative phase—a trait of high agronomic relevance that can increase fruiting frequency within a growing season and facilitate more synchronized fruit set (Figure 3H–P). These architectural shifts are also expected to influence fruit size and reproductive load by altering hormonal signalling and resource distribution between vegetative and reproductive tissues—consistent with findings in tomato where compact and determinate genotypes displayed improved fruit yield and resource‐use efficiency through reduced apical dominance and balanced hormone gradients (Park et al. 2014; Kwon et al. 2020).

The improvements in fruit yield per plant observed in the double determinate line further support the conclusion that compact determinate plants efficiently channel assimilates toward reproductive sinks, aligning with *SP* and *SP5G*‐mediated yield improvement through improved photosynthate partitioning toward tomato (Pnueli et al. 1998; Soyk, Lemmon, et al. 2017; Lemmon et al. 2018). Regarding day‐length sensitivity, wild tomato ( *Solanum pennellii* ) flowers after approximately 12 leaves under long‐day conditions—a photoperiod sensitivity that was reduced during domestication through selection for reduced *SP5G* activity (Soyk, Müller, et al. 2017). Similarly, WT BGB flowers after 10–12 leaves under long‐day conditions, suggesting comparable day‐length sensitivity. Loss of *LbSP5G1* function in edited lines promoted faster flowering with fewer leaves before the first inflorescence, consistent with the mitigation of day‐length sensitivity observed in *SP5G* null mutant tomato lines under both long‐day and short‐day conditions (Soyk, Müller, et al. 2017). This reduction in photoperiod sensitivity has important practical implications for expanding goji cultivation into regions with varying light regimes and enabling more predictable flowering and fruit set across diverse production environments.

One notable distinction between goji berry and other Solanaceous edited lines is the emergence of terminal spikes at the shoot apex in single and double edited goji plants—a phenotype not reported in equivalent *SP* mutants in tomato or ground cherry. This observation likely reflects species‐specific differences in inflorescence architecture and meristem organization between *Lycium* and other Solanaceous genera—consistent with the broader principle that orthologous PEBP gene mutations can produce phenotypically distinct outcomes across species due to differences in genetic background, regulatory context, and developmental architecture (Kwon et al. 2020). The mechanistic basis of terminal spike formation in goji berry represents an intriguing open question—potentially involving species‐specific interactions between *SP*‐type proteins and inflorescence identity regulators, and warrants dedicated investigation in future studies. Understanding this phenomenon could reveal novel regulatory genes in goji inflorescence development and may provide additional targets for fine‐tuning shoot architecture in this economically important crop.

### 4.4 *LbSP1* ‐ and *LbSP5G1* ‐Centred Transcriptional Networks Integrate Multiple Regulatory Modules to Control Meristem Fate and Shoot Determinacy in BGB

Transcriptome analysis of SAM and S1 fruit tissues from WT and gene‐edited lines revealed that disruption of *LbSP1* and *LbSP5G1* leads to extensive reprogramming of flowering regulatory networks in BGB, promoting a shift toward determinate growth (Figures S13–S15), characterized by coordinated downregulation of key flowering regulators, including MADS‐box, SPL, and WUS genes, along with reduced expression of photosynthesis‐related genes, consistent with decreased meristem activity (Figure S14). The downregulation of photosynthetic genes in the SAM is consistent with a shift in resource allocation toward reproductive development (Zheng et al. 2022), while the reduced expression of WUS aligns with its established role in maintaining stem cell populations (Mayer et al. 1998; Ma et al. 2019). RT‐qPCR analysis further supported these findings, confirming downregulation of all tested genes in the SAM of edited lines, while a subset including MYB1–MYB3, bHLH1, WUS1‐WUS2, and DELLA1 displayed increased expression in S1 fruits, indicating tissue‐specific transcriptional reprogramming following *LbSP1* and *LbSP5G1* disruption (Figure S16). The coordinated expression changes observed for B3‐1/ARF and DELLA1 suggest their involvement in transcriptional networks associated with meristem regulation, consistent with established roles of auxin and gibberellin pathways in flowering through ARF‐ and DELLA‐mediated mechanisms (Yamaguchi et al. 2013).

Co‐expression network analysis positioned *LbSP1* and *LbSP5G1* as central hubs within modules enriched for multiple TFs families, including MYB, WRKY, AP2/ERF, bHLH, TCP and ARFs, suggesting their roles in integrating multiple regulatory pathways, consistent with previous WGCNA‐based studies of flowering regulation (Matías‐Hernández et al. 2016; Zheng et al. 2022). Additional hub genes including *MYB*, *AP2/ERFs*, *WUS* and *WRKY*, along with auxin‐ and gibberellin‐related genes including ARFs and DELLA, were identified in the grey, black, and brown modules, suggesting their potential involvement in flowering regulation (Figure S15E–G). Previous studies have demonstrated that *WRKY75* promotes flowering via *FT* activation (Zhang et al. 2018). While other TF families, including no apical meristem (NAC‐NAM), basic leucine zipper (bZIP), and homeobox proteins, also contribute to flowering regulation and stress responses (Jakoby et al. 2002; Minh‐Thu et al. 2018), collectively highlighting the complexity of transcriptional control underlying shoot determinacy in goji berry.

**FIGURE 5:**
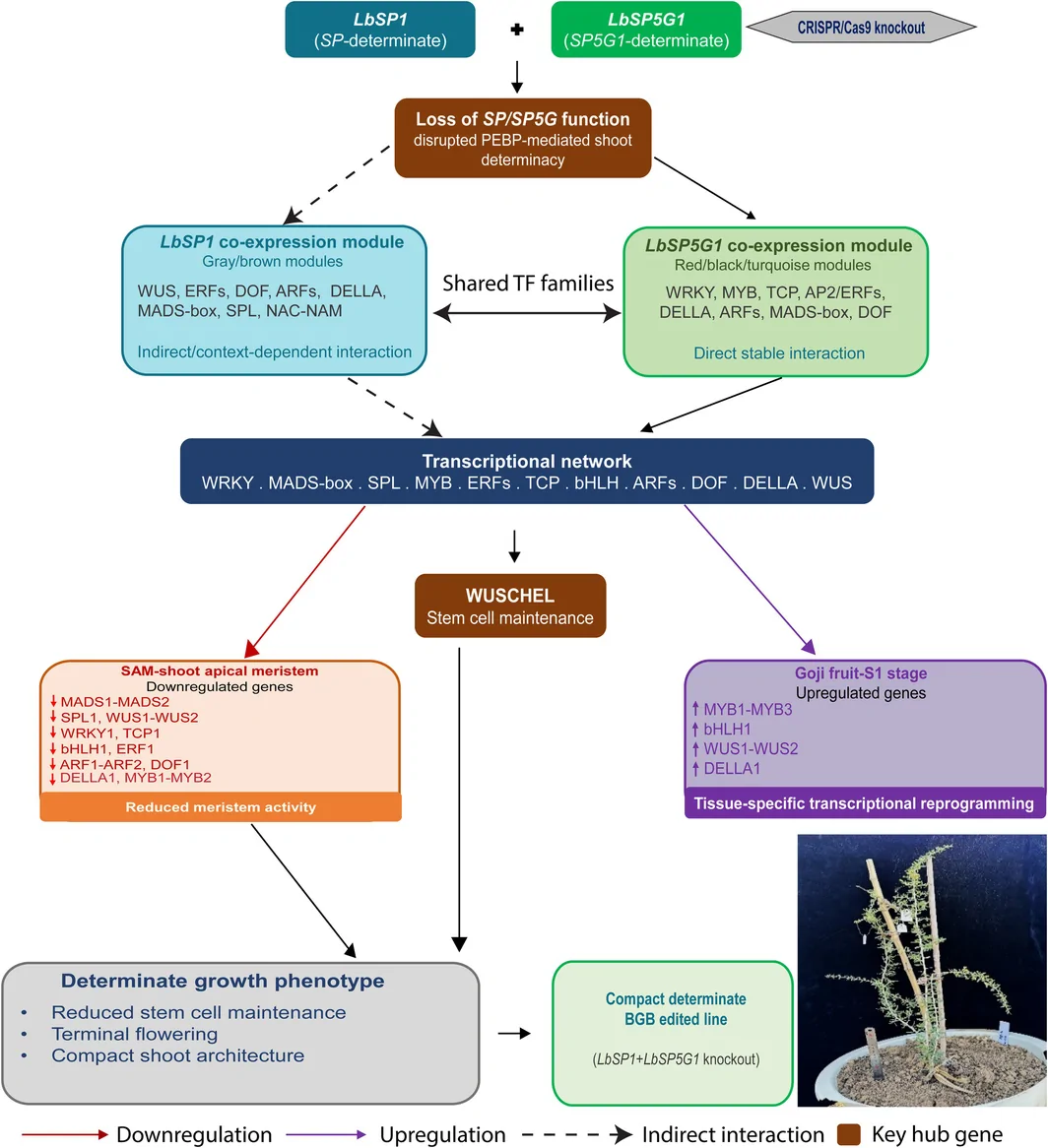
Proposed regulatory framework for *LbSP1*‐ and *LbSP5G1*‐mediated control of meristem determinacy and shoot architecture in *L. ruthenicum*. CRISPR/Cas9‐mediated knockout of *LbSP1* and *LbSP5G1* disrupts PEBP‐mediated shoot determinacy—altering the balance of *SP/SP5G*‐type regulatory activity and triggering extensive transcriptional reprogramming. *LbSP5G1* acts as a direct interaction hub, forming stable protein–protein interactions with DELLA1, MADS‐box, MYB, and DOF1 under stringent Y2H conditions, and additionally interacting with SPL, ERFs, WUS, TCP, and ARFs as detected by LCI assays in tobacco leaves. In contrast, *LbSP1* exhibits context‐dependent or indirect interactions behaviour, with moderate LCI signals detected for DELLA1, ERFs, B3/ARF, MADS‐box, WUS, TCP and DOF1, but no detectable interactions under stringent Y2H conditions. The two co‐expression modules identified by WGCNA—the *LbSP1*‐associated module (grey/brown; WUS, ERF, DOF, ARFs, DELLA, MADS‐box, SPL, NAC‐NAM) and the *LbSP5G1*‐associated module (red/black/turquoise; WRKY, MYB, TCP, ARFs, AP2/ERF, DELLA, MADS‐box, DOF)—converge on a broader transcriptional regulatory network comprising WRKY, MADS‐box, SPL, MYB, ERF, TCP, bHLH, ARFs, DOF, DELLA, and WUS transcription factors. Disruption of *LbSP1* and *LbSP5G1* leads to coordinated downregulation of key flowering regulators, including MADS‐box, SPL, WUS, WRKY, TCP, bHLH, ERF, ARFs, DOF, DELLA and MYB, in the shoot apical meristem (SAM), reducing meristem activity and promoting a determinate growth phenotype. Tissue‐specific transcriptional reprogramming was observed, with genes including MYB1‐MYB3, bHLH1, WUS1‐WUS2 and DELLA1, showing contrasting upregulation in S1 goji fruit tissues. These coordinated changes collectively promote reduced stem cell maintenance, terminal flowering, and a compact shoot architecture. Solid arrows indicate direct protein–protein interactions confirmed by Y2H; dashed arrows indicate indirect or context‐dependent interactions detected by LCI; line with arrowheads indicate transcriptional regulatory flow and expression changes; red downward arrows indicate gene downregulation in SAM; purple upward arrows indicate tissue‐specific upregulation in S1 fruit.

Protein–protein interaction assays provided mechanistic insights into the functional divergence between *LbSP1* and *LbSP5G1*. *LbSP5G1* exhibited stable interactions with DELLA1, MADS‐box, MYB, and DOF1 under stringent Y2H conditions and additionally showed strong luminescence signals with SPL, DELLA1, MADS‐box, WUS, MYB, and DOF1 in LCI assays, indicating its direct involvement in transcriptional regulatory complexes (Figure 4). Given the established roles of these TFs in floral development (Yanagisawa 2002; Fornara et al. 2009, 2010; Dubos et al. 2010), *LbSP5G1* likely functions as a direct molecular bridge linking PEBP signalling to downstream transcriptional modules. In contrast, *LbSP1* did not exhibit detectable interactions under stringent yeast conditions but showed moderate in planta interaction signals with DELLA, B3‐1/ARFs, MADS‐box, ERFs, WUS, TCP and DOF1, suggesting that it operates through context‐dependent or transient interactions that may require plant‐specific cofactors. Such behaviour is consistent with previous reports that SP/TFL1‐like proteins often function through indirect mechanisms or as components of multiprotein complexes (Bradley et al. 1997; Hanano and Goto 2011). Together, these interaction profiles establish a clear functional divergence—*LbSP5G1* acts as a direct interaction hub within the flowering regulatory network while *LbSP1* contributes through indirect or context‐dependent regulatory modulation (Figures 4 and S15). Collectively, these results support a refined regulatory framework in which *LbSP1* and *LbSP5G1* act as central components of the PEBP‐centred network that integrates multiple TFs modules, including MYB–WRKY–AP2/ERF–TCP–MADS, to control meristem fate and shoot determinacy in goji berry (Figure 5). Disruption of these genes alters the balance of PEBP‐mediated regulation, leading to downstream suppression of MADS‐box, SPL, MYB, and WUS pathways, thereby promoting floral determinacy and reducing meristem maintenance—a multi‐layered regulatory mechanism broadly conserved across angiosperms (Moraes et al. 2019; Jin et al. 2021). This coordinated regulation ultimately drives the transition from indeterminate to determinate growth, providing a mechanistic basis for the compact shoot architecture observed in gene‐edited goji lines (Figure 5).

### 4.5 Future Directions

Building on the functional genomic framework established in this study—several important directions emerge for advancing both the mechanistic understanding and practical application of *LbSP1* and *LbSP5G1* in goji berry improvement. Given the presence of additional *SP‐like* gene copies in the *L. barbarum* genome—beyond the two characterized here—future studies investigating potential functional redundancy or specialization among these paralogs could reveal additional regulatory layers controlling meristem fate and flowering. Multiplexed CRISPR/Cas9 approaches targeting combinations of *SP‐like* paralogs alongside *LbSP1* and *LbSP5G1* may further enhance shoot compactness and synchronize fruit development beyond what was achieved in the present single and double mutant lines. Further molecular dissection of the regulatory architecture governing *LbSP1* and *LbSP5G1* expression, including comparative analysis of their cis‐regulatory landscapes between indeterminate and determinate accessions, may clarify whether promoter‐level variation contributes to the natural differences in shoot determinacy observed in goji berry. Complementing this, future genetic studies incorporating whole‐genome resequencing of contrasting accessions, allelic variation analysis at *LbSP1* and *LbSP5G1* loci, and genetic mapping in segregating populations would provide the classical genetic evidence necessary to fully establish the contribution of these genes to natural growth habit variation in *Lycium*, representing an important next step toward a complete molecular genetic framework for shoot architecture in goji berry. From an agronomic perspective, the determinate and compact growth habit observed in the edited lines, together with the reduced number of leaves to the first inflorescence and more synchronized flowering, suggests a potential trend toward improved flowering and fruit‐set synchrony that warrants quantitative evaluation under field conditions. Comprehensive field‐based assessment of the edited lines across contrasting photoperiod and temperature environments will be essential to evaluate agronomic stability, yield performance, and environmental adaptability, and to develop environment‐specific ideotypes suitable for mechanized harvesting and consistent fruit production across goji cultivation zones. Additionally, stacking *LbSP1* and *LbSP5G1* mutations with fruit size regulators such as *CLV3* (Muños et al. 2011; Li, Yang, et al. 2018), and *YABBY2* (Cong et al. 2008) may enable simultaneous control of meristem determinacy, organ size, and fruit development, potentially generating cultivars with both architectural compactness and improved fruit quality traits.

Interestingly, preliminary observations in this study indicated potential changes in anthocyanin accumulation in the edited lines, suggesting a possible link between *SP/SP5G*‐mediated meristem regulatory pathways and secondary metabolism. Future investigations integrating metabolomic profiling and transcriptional analysis of anthocyanin biosynthetic genes may help elucidate whether PEBP‐mediated pathways indirectly influence pigment biosynthesis, with implications for the nutritional and commercial value of goji berry cultivars. Furthermore, SEM analysis at defined developmental stages in edited lines will be essential to directly validate meristem alterations underlying the observed shoot architecture phenotypes and to investigate the mechanistic basis of the novel terminal spike formation observed in *LbSP1* and *LbSP1* + *LbSP5G1* edited lines. Collectively, these future investigations will facilitate the translation of CRISPR‐based modifications into practical breeding strategies, advancing the development of next‐generation compact, synchronized‐flowering, high‐yield goji cultivars optimized for modern mechanized production systems.

## 5 Conclusion

In conclusion, this study provides the first functional genomic evidence that PEBP‐mediated shoot determinacy in goji berry is governed by two conserved regulators (*LbSP1* and *LbSP5G1*), whose coordinated disruption is sufficient to reprogram an indeterminate sprawling growth habit into a compact, synchronized, and improved‐yielding determinate form with direct agronomic value. Beyond demonstrating causality through CRISPR/Cas9 functional characterization, the transcriptomic and molecular interaction evidence presented here collectively defines a regulatory architecture in which *LbSP1* and *LbSP5G1* occupy distinct but complementary positions within a PEBP‐centred network—*LbSP5G1* as a direct interaction hub and *LbSP1* as a context‐dependent modulator, together orchestrating the balance between meristem maintenance and reproductive commitment in *Lycium*. This study not only advances the mechanistic understanding of shoot architecture regulation in an underexplored but economically important berry crop—it also establishes the molecular and technical foundations upon which targeted breeding of next‐generation goji cultivars can be built.

## Author Contributions

Y. Wang and F.R. conceived and designed the research; F.R. wrote the original draft manuscript; F.R., H.L., and Y.M. collected the samples for RNA sequencing and microscopic examination, F.R. performed the RNA sequencing data analysis, software, formal analysis, interpretation, and identified the specific genes; H.L. performed the microscopic examination of goji shoot architecture, F.R. performed bioinformatic analysis, designed the gRNAs, and constructed the CRISPR/Cas9 single and multiplexed vectors; F.R. performed the genetic transformation of goji berries, identification of positive lines, and phenotypic traits quantification, subsequent analysis, and RT‐qPCR; F.R. and Y. Wu designed and performed protein – protein interaction assays; Y. Wang provided project supervision and funding acquisition; F.R., Y. Wang, C.Y., S.Z., Y.Z. reviewed and edited the manuscript. All the authors have read and approved the final manuscript.

## Funding

This study was supported by the National Natural Science Foundation of China (32170389, 32570435 and 32370399), Guangdong Science and Technology Plan Project (2023B1212060046), Strategic Priority Research Program of the Chinese Academy of Sciences (XDA0440000), and the Key Technologies R & D Program of Guangdong Province (2022B1111230001).

## Conflicts of Interest

The authors declare no conflicts of interest.

## Supporting information


**Figure S1:** Representative photographs and microscopic images of shoot apex samples collected from indeterminate and determinate *Lycium barbarum* plants for morphological characterization and transcriptomic analysis.
**Figure S2:** Vector maps of single and multiplexed CRISPR/Cas9 constructs and overview of *Lycium ruthenicum* genetic transformation.
**Figure S3:** Quality assessment and reproducibility analysis of RNA sequencing data from three different shoot apex sample groups of *L. barbarum*.
**Figure S4:** Differential expression analysis of genes associated with flowering regulation across three shoot apex pairwise comparisons in *L. barbarum*.
**Figure S5:** GO and KEGG enrichment analysis of up‐ and down‐regulated DEGs in the N versus TA comparison in *L. barbarum* shoot apices.
**Figure S6:** GO and KEGG enrichment analysis of upregulated and downregulated DEGs in N versus TB and TA versus TB comparisons in *L. barbarum* shoot apices.
**Figure S7:** Hierarchical clustering analysis of differentially expressed genes (DEGs) across three shoot apex sample groups in *L. barbarum*.
**Figure S8:** RT‐qPCR validation of 15 candidate genes selected from transcriptome analysis of shoot apex samples (N, TA, and TB) in *L. barbarum*.
**Figure S9:** Linear regression analysis comparing RNA‐seq log2FC values and RT‐qPCR log2FC values for the 15 validated candidate genes across three pairwise comparisons (N vs. TA, N vs. TB, and TA vs. TB).
**Figure S10:** Multiple sequence alignment and phylogenetic analysis of *LbSP1* and *LbSP5G1* with TFL1/SP family members from *Arabidopsis thaliana*, *Solanum lycopersicum*, and *Oryza sativa*.
**Figure S11:** Molecular validation of CRISPR/Cas9‐mediated genome editing and transgene‐free confirmation in *L. ruthenicum* edited lines.
**Figure S12:** Expression validation of *LbSP1* and *LbSP5G1* in CRISPR/Cas9‐edited *L. ruthenicum* lines.
**Figure S13:** Transcriptomic analysis of SAM and S1 fruit tissues from WT and CRISPR/Cas9 edited *L. ruthenicum* lines.
**Figure S14:** Hierarchically clustering heatmaps showing expression profiles of GO and KEGG enriched DEGs associated with flowering regulatory pathways in SAM and S1 fruit tissues of WT and CRISPR/Cas9‐edited *L. ruthenicum* lines.
**Figure S15:** Weighted gene co‐expression network analysis (WGCNA) of DEGs from SAM and S1 fruit tissues of WT and *LbSP1/LbSP5G*1‐edited *L. ruthenicum* lines.
**Figure S16:** RT‐qPCR validation of co‐expression network hub genes associated with flowering regulation and meristem development in SAM and S1 fruit tissues of WT and CRISPR/Cas9‐edited *L. ruthenicum* lines.

**Table S1:** Statistics of sequencing data quality and clean reads, clean bases of nine shoot apices samples.
**Table S2:** Statistics of sequencing data quality and clean reads, clean bases of 16 S1 and SAM sample of four genotypes.
**Table S3:** List of primers used for CRISPR/Cas9 vector construction and gene identification.
**Table S4:** List of primers used for RT‐qPCR.
**Table S5:** List of primers used for Y2H and LUC assays.
**Table S6:** Clean reads mapping with reference genome of goji berry.
**Table S7:** GO functional enrichment of N versus TA DEGs.
**Table S8:** Expression values of 44 significant DEGs for all nine samples of shoot apices.
**Table S9:** CRISPR gene edited single and double mutant transgenic lines with gene editing and transformation efficiency.
**Table S10:** Off‐target site prediction using online tool for *LbSP1* and *LbSP5G1*.
**Table S11:** Raw and mean phenotypic data of seven traits corresponding to the wild type, single and double CRISPR/Cas9 gene edited transgenic lines.
**Table S12:** Top 25 GO enriched pathways for differentially expressed genes in the biological process (BP) based on the comparison between W‐S1 versus CRLbSP1‐S1, W‐S1 versus CRLbSP5G1‐S1, W‐S1 versus 2gCR‐S1.
**Table S13:** Top 25 GO enriched pathways for differentially expressed genes in the biological process (BP) based on the comparison between W‐SAM versus CRLbSP1‐SAM, W‐SAM versus CRLbSP5G1‐SAM, W‐SAM versus 2gCR‐SAM.

## Data Availability

The raw sequence data reported in this paper have been deposited in the Genome Sequence Archive (Zhang et al. 2025), in National Genomics Data Center (Bao et al. 2025), China National Center for Bioinformation/Beijing Institute of Genomics, Chinese Academy of Sciences (GSA: CRA035881) that are publicly accessible at https://ngdc.cncb.ac.cn/gsa.
