# Supplementary material for: CRISPR/Cas9‐Mediated Knockout of LbSP1 and LbSP5G1 Reveals Efficient Customization of Shoot Architecture in Black Goji Berry
Source: Plant Biotechnol J. 2026 Sep 2;24(11):5868–90. doi: 10.1111/pbi.70753 (PMC13536850; doi:10.1111/pbi.70753)
Supplement: Supplementary file 1 [file PBI-24-5868-s002.pdf]

Supplementary information

CRISPR/Cas9-Mediated Knockout of *LbSP1* and *LbSP5G1* Reveals Efficient Customization of Shoot Architecture in Black Goji Berry

Supplementary Figures

FIGURE S1 | Representative photographs and microscopic images of shoot apex samples collected from indeterminate and determinate *Lycium barbarum* plants for morphological characterization and transcriptomic analysis.

FIGURE S2 | Vector maps of single and multiplexed CRISPR/Cas9 constructs and overview of *Lycium ruthenicum* genetic transformation.

FIGURE S3 | Quality assessment and reproducibility analysis of RNA sequencing data from three different shoot apex sample groups of *L. barbarum*.

FIGURE S4 | Differential expression analysis of genes associated with flowering regulation across three shoot apex pairwise comparisons in *L. barbarum*.

FIGURE S5 | GO and KEGG enrichment analysis of up- and down-regulated DEGs in the N versus TA comparison in *L. barbarum* shoot apices.

FIGURE S6 | GO and KEGG enrichment analysis of upregulated and downregulated DEGs in N versus TB and TA versus TB comparisons in *L. barbarum* shoot apices.

FIGURE S7 | Hierarchical clustering analysis of differentially expressed genes (DEGs) across three shoot apex sample groups in *L. barbarum*.

FIGURE S8 | RT-qPCR validation of fifteen candidate genes selected from transcriptome analysis of shoot apex samples (N, TA, and TB) in *L. barbarum*.

FIGURE S9 | Linear regression analysis comparing RNA-seq $\log_2$FC values and RT-qPCR $\log_2$FC values for the fifteen validated candidate genes across three pairwise comparisons (N vs. TA, N vs. TB, and TA vs. TB).

FIGURE S10 | Multiple sequence alignment and phylogenetic analysis of *LbSP1* and *LbSP5G1* with TFL1/SP family members from *Arabidopsis thaliana*, *Solanum lycopersicum*, and *Oryza sativa*.

FIGURE S11 | Molecular validation of CRISPR/Cas9-mediated genome editing and transgene-free confirmation in *L. ruthenicum* edited lines.

FIGURE S12 | Expression validation of *LbSP1* and *LbSP5G1* in CRISPR/Cas9-edited *L. ruthenicum* lines.

FIGURE S13 | Transcriptomic analysis of SAM and S1 fruit tissues from WT and CRISPR/Cas9-edited *L. ruthenicum* lines.

FIGURE S14 | Hierarchically clustering heatmaps showing expression profiles of GO and KEGG-enriched DEGs associated with flowering regulatory pathways in SAM and S1 fruit tissues of WT and CRISPR/Cas9-edited *L. ruthenicum* lines.

FIGURE S15 | Weighted gene co-expression network analysis (WGCNA) of DEGs from SAM and S1 fruit tissues of WT and *LbSP1/LbSP5G1*-edited *L. ruthenicum* lines.

FIGURE S16 | RT-qPCR validation of co-expression network hub genes associated with flowering regulation and meristem development in SAM and S1 fruit tissues of WT and CRISPR/Cas9-edited *L. ruthenicum* lines.

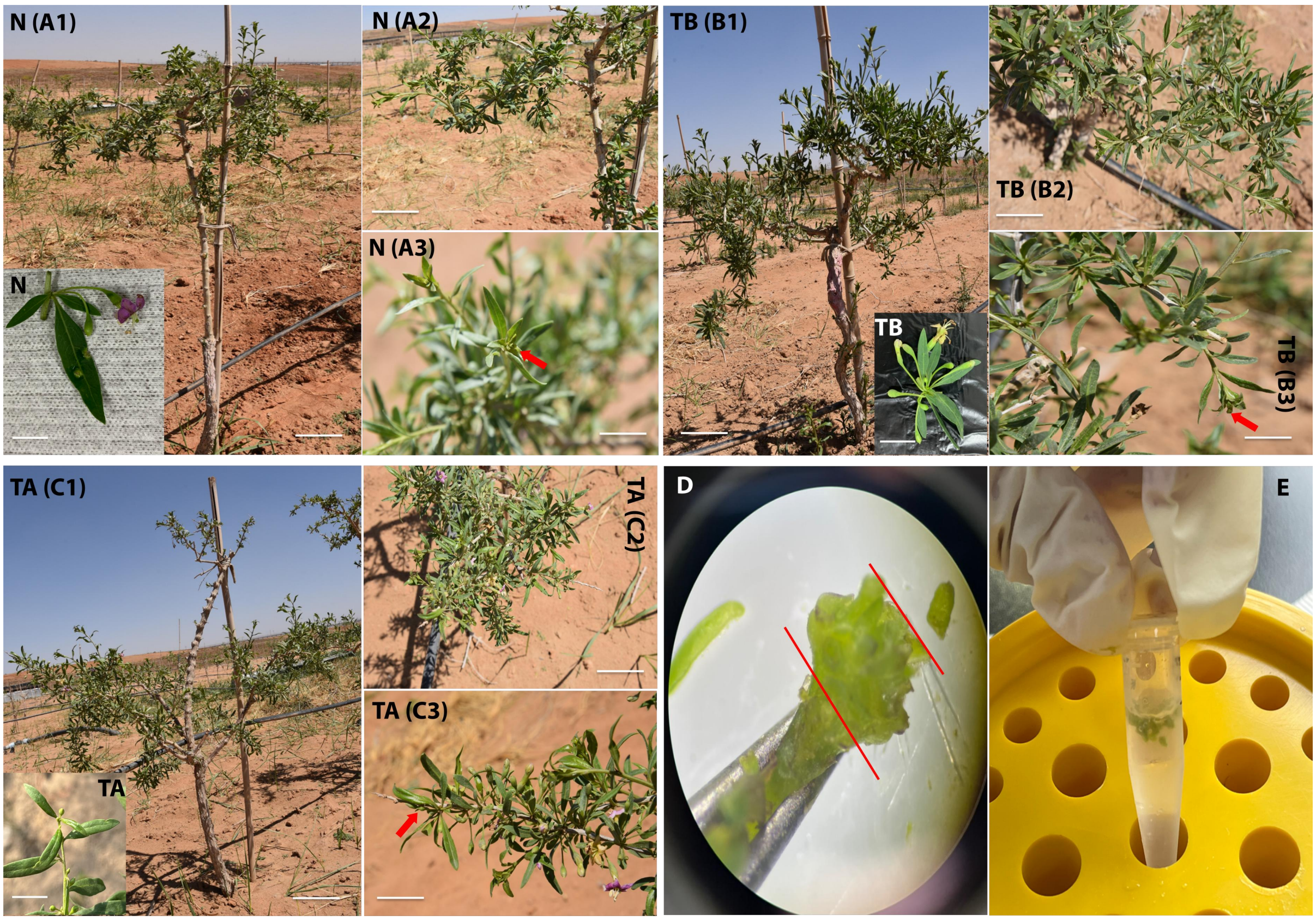

FIGURE S1 | Representative photographs and microscopic images of shoot apex samples collected from indeterminate and determinate *Lycium barbarum* plants for morphological characterization and transcriptomic analysis. (**A1-A3**) Non-terminal shoot apices of indeterminate individual plants (N). **(B1-B3)** Terminal shoot apices of determinate individual plants before apical termination (TB). **(C1-C3)** Terminal shoot apices of determinate individual plants after apical inflorescence initiation at the apical meristem (TA). **(D)** Microscopic image of a representative shoot apex sample of approximately 2 mm, the tissue size used for SEM and RNA extraction. **(E)** Shoot apex sample preparation prior to RNA sequencing, showing dissection and immediate liquid nitrogen preservation. Red arrows indicate the shoot apical meristem. Scale bars: 5cm and 1cm (in pictures).

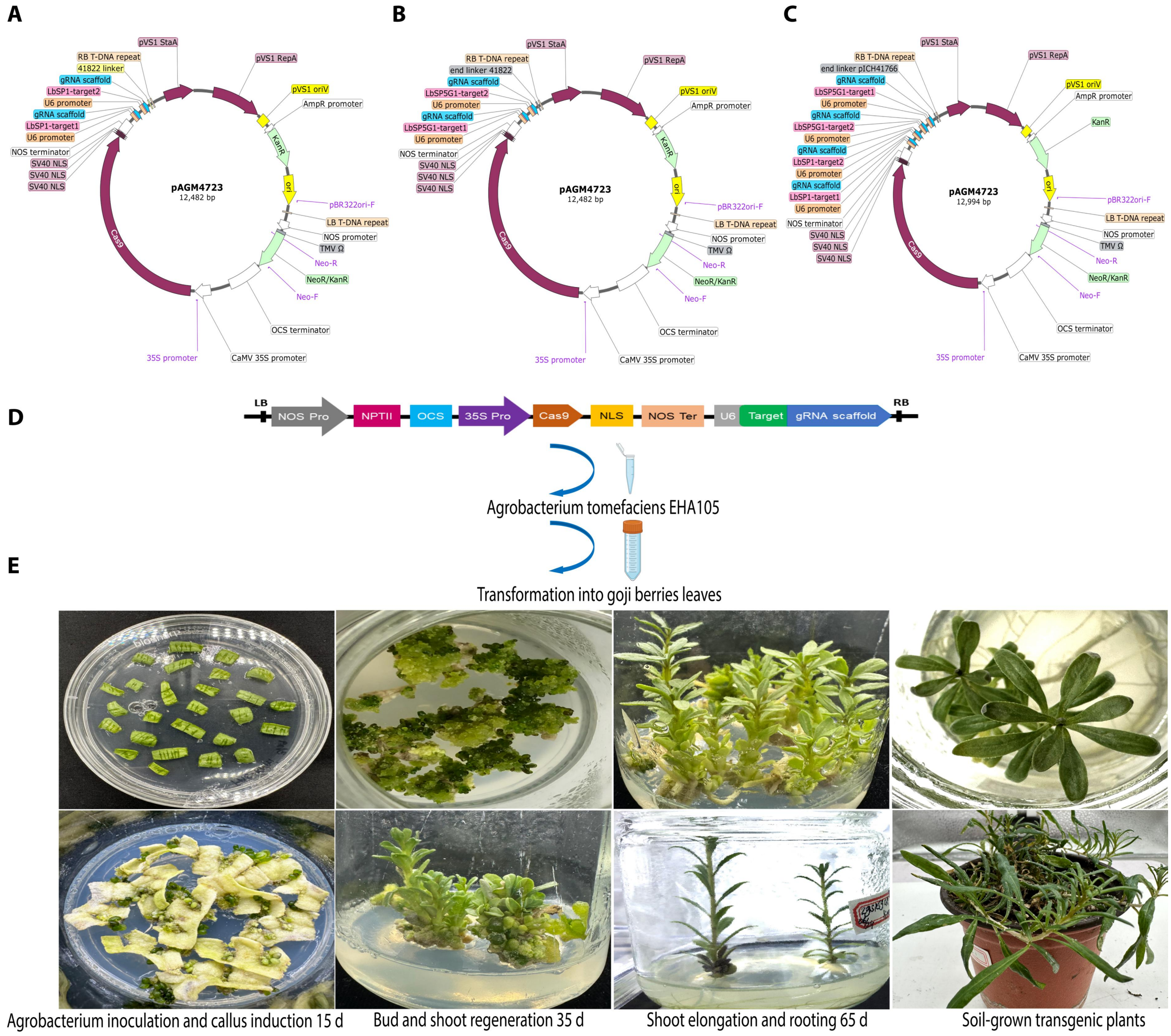

FIGURE S2 | Vector maps of single and multiplexed CRISPR/Cas9 constructs and overview of *Lycium ruthenicum* genetic transformation. **(A)** Vector map of the single CRISPR/Cas9 construct targeting *LbSP1*. **(B)** Vector map of the single CRISPR/Cas9 construct targeting *LbSP5G1*. **(C)** Vector map of the multiplexed CRISPR/Cas9 construct simultaneously targeting *LbSP1+SP5G1*. **(D)** Schematic diagram of the CRISPR/Cas9 expression cassette and *Agrobacterium*-mediated genetic transformation of black goji berry. **(E)** Representative photographs illustrating the sequential stages of goji berry genetic transformation: *Agrobacterium* inoculation and callus induction (15 d), shoot bud initiation and regeneration (35 d), shoot elongation and rooting (65 d), and establishment of soil-grown transgenic plants.

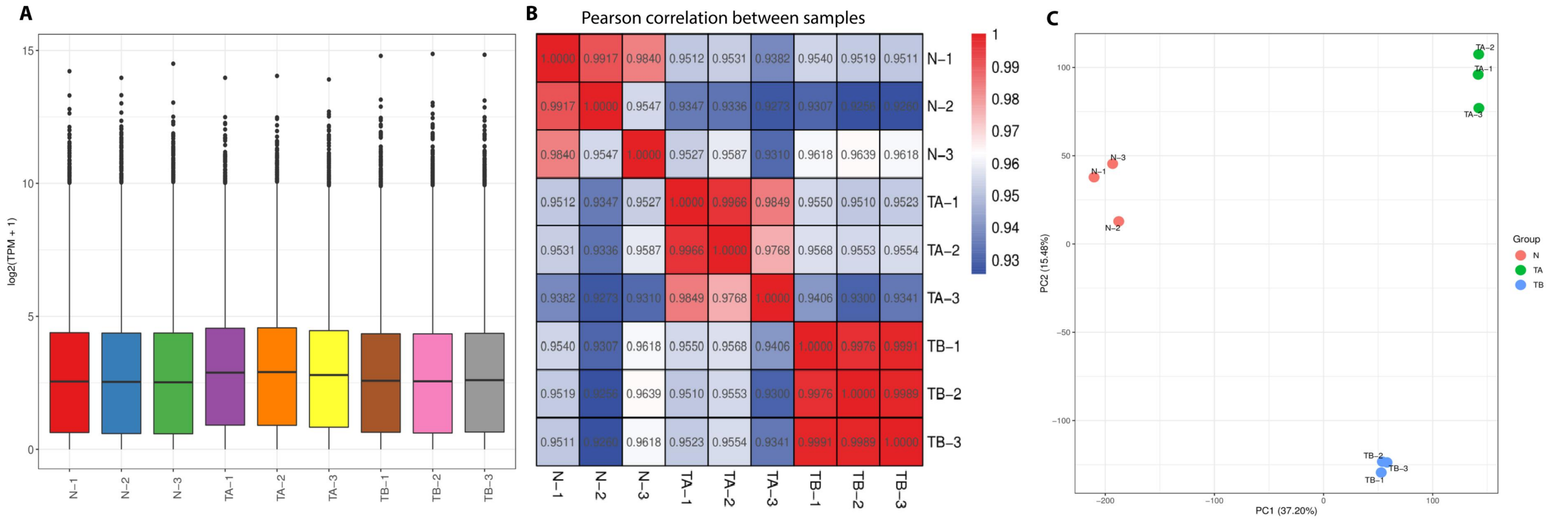

FIGURE S3 | Quality assessment and reproducibility analysis of RNA sequencing data from three different shoot apex sample groups of *L. barbarum*. **(A)** Box plots showing the $\log_2$-transformed FPKM distribution across all nine samples (N1-N3, TA1-TA3, TB1-TB3, confirming uniform sequencing depth and data quality. (**B**) Pearson correlation heatmap showing pairwise correlation coefficients among the nine shoot apex samples, demonstrating high within group reproducibility. **(C)** Principal component analysis (PCA) biplot representing sample clustering by developmental group, with PC1 and PC2 explaining the major sources of transcriptomic variance.

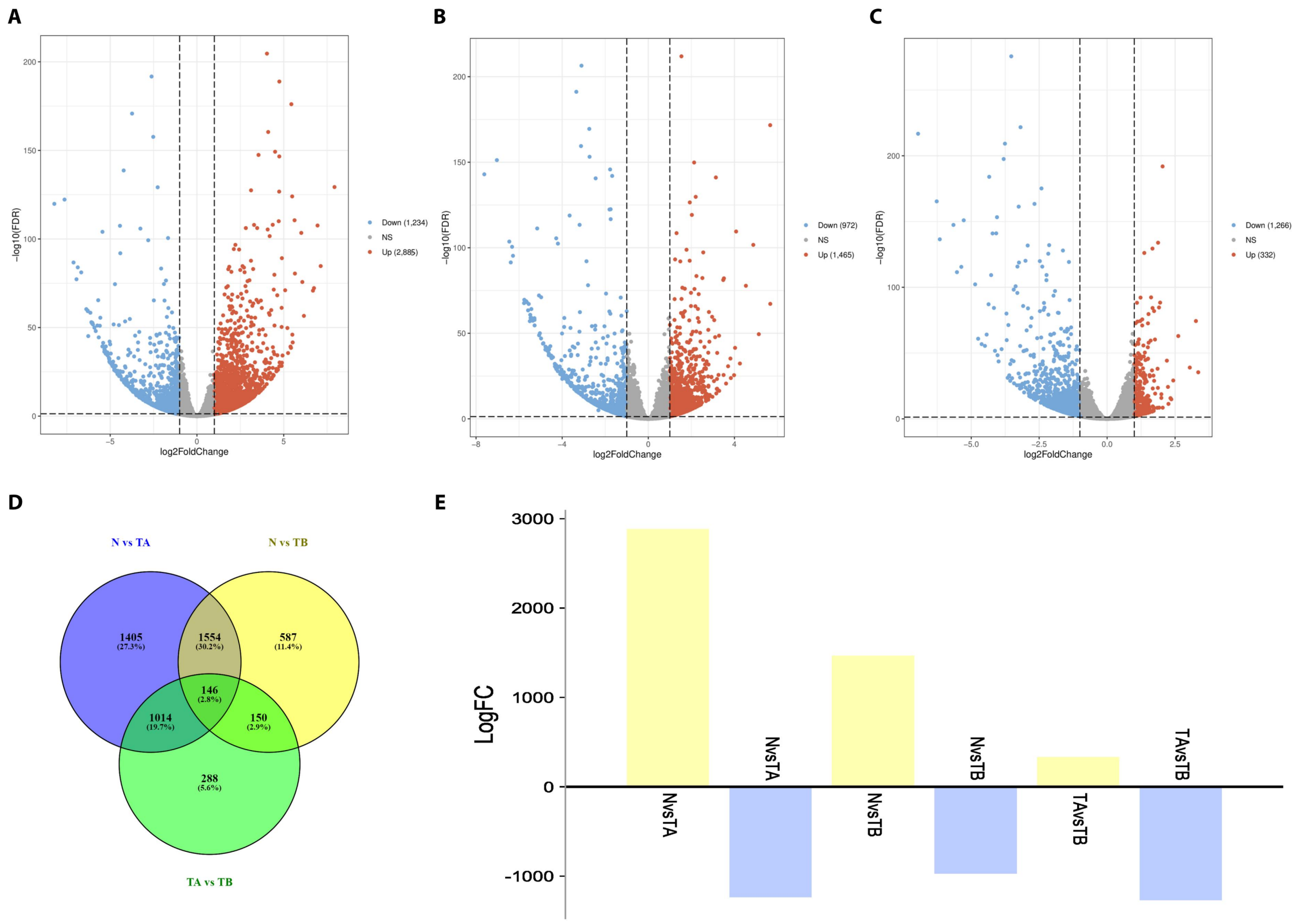

FIGURE S4 | Differential expression analysis of genes associated with flowering regulation across three shoot apex pairwise comparisons in *L. barbarum*. **(A-C)** Volcano plots of all expressed DEGs in N vs. TA (A), N vs. TB (B), and TA vs. TB (C), with $\log_2$FC values plotted against $-\log_{10}$ (FDR-adjusted *p*-values). Red and blue dots represent significantly up- and down-regulated DEGs ($|\log_2\text{FC}| \geqslant 1$, FDR $\leqslant$ 0.05), and grey dots represent non-significant genes. **(D)** Venn diagram showing the number of common and unique DEGs across the three pairwise comparisons. **(E)** Summary of up- and down-regulated DEGs in each comparison: N vs. TA (2,885 upregulated, 1,234 downregulated), N vs. TB (1,465 upregulated, 972 downregulated), TA vs. TB (332 upregulated, 1,266 downregulated).

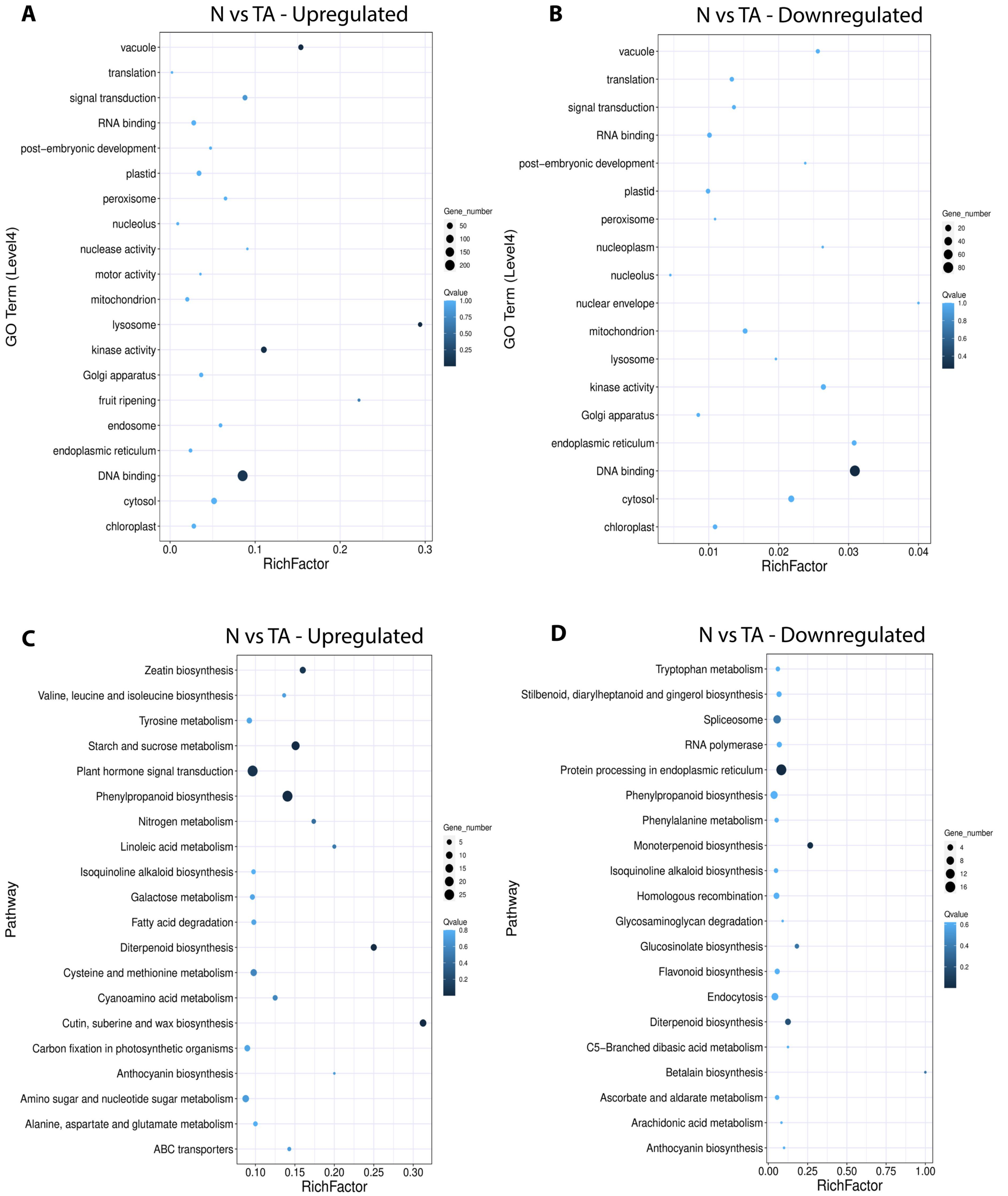

FIGURE S5 | GO and KEGG enrichment analysis of up- and down-regulated DEGs in the N versus TA comparison in *L. barbarum* shoot apices. **(A, B)** Top 30 significantly enriched GO terms across biological process (BP), molecular function (MF), and cellular component (CC) categories for upregulated (A) and downregulated (B) DEGs in N vs. TA ($p \leqslant 0.05$). **(C, D)** Top 20 significantly enriched KEGG pathways for upregulated (C) and downregulated (D) DEGs in N vs. TA ($p \leqslant 0.05$). The x-axis represents the gene ratio (proportion of DEGs mapped to each pathway), the y-axis represents the significance level (*Q*-value; dark blue: more significant; light blue: less significant), and dot size indicates the number of genes enriched in each term or pathway.

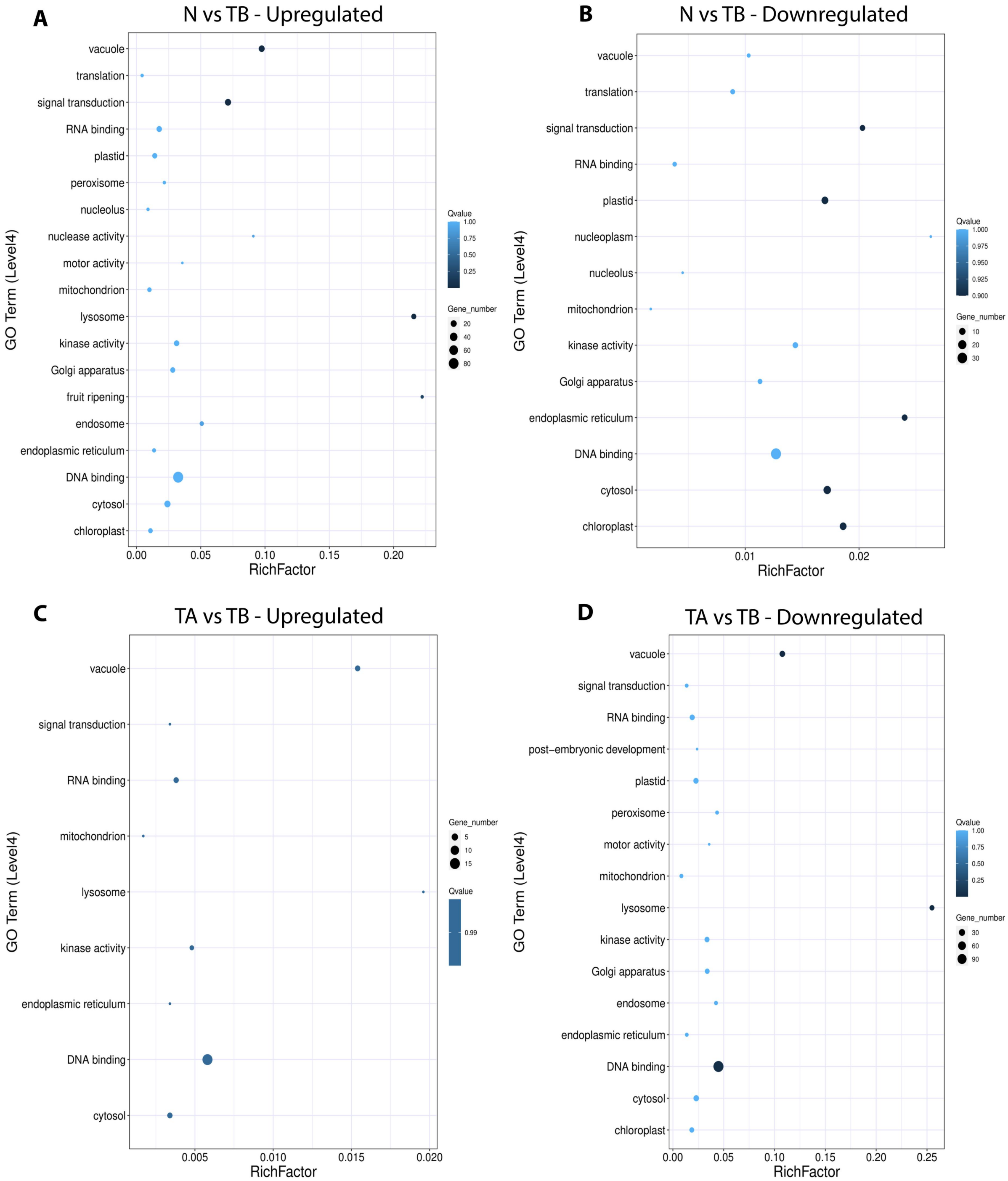

A
N vs TB - Upregulated
vacuole
translation
signal transduction
RNA binding
plastid
peroxisome
nucleolus
nuclease activity
motor activity
mitochondrion
lysosome
kinase activity
Golgi apparatus
fruit ripening
endosome
endoplasmic reticulum
DNA binding
cytosol
chloroplast
GO Term (Level4)
RichFactor
0.00 0.05 0.10 0.15 0.20
Qvalue
1.00 0.75 0.50 0.25
Gene_number
20 40 60 80
B
N vs TB - Downregulated
vacuole
translation
signal transduction
RNA binding
plastid
nucleoplasm
nucleolus
mitochondrion
kinase activity
Golgi apparatus
endoplasmic reticulum
DNA binding
cytosol
chloroplast
GO Term (Level4)
RichFactor
0.01 0.02
Qvalue
1.000 0.975 0.950 0.925 0.900
Gene_number
10 20 30
C
TA vs TB - Upregulated
vacuole
signal transduction
RNA binding
mitochondrion
lysosome
kinase activity
endoplasmic reticulum
DNA binding
cytosol
GO Term (Level4)
RichFactor
0.005 0.010 0.015 0.020
Gene_number
5 10 15
Qvalue
0.99
D
TA vs TB - Downregulated
vacuole
signal transduction
RNA binding
post−embryonic development
plastid
peroxisome
motor activity
mitochondrion
lysosome
kinase activity
Golgi apparatus
endosome
endoplasmic reticulum
DNA binding
cytosol
chloroplast
GO Term (Level4)
RichFactor
0.00 0.05 0.10 0.15 0.20 0.25
Qvalue
1.00 0.75 0.50 0.25
Gene_number
30 60 90

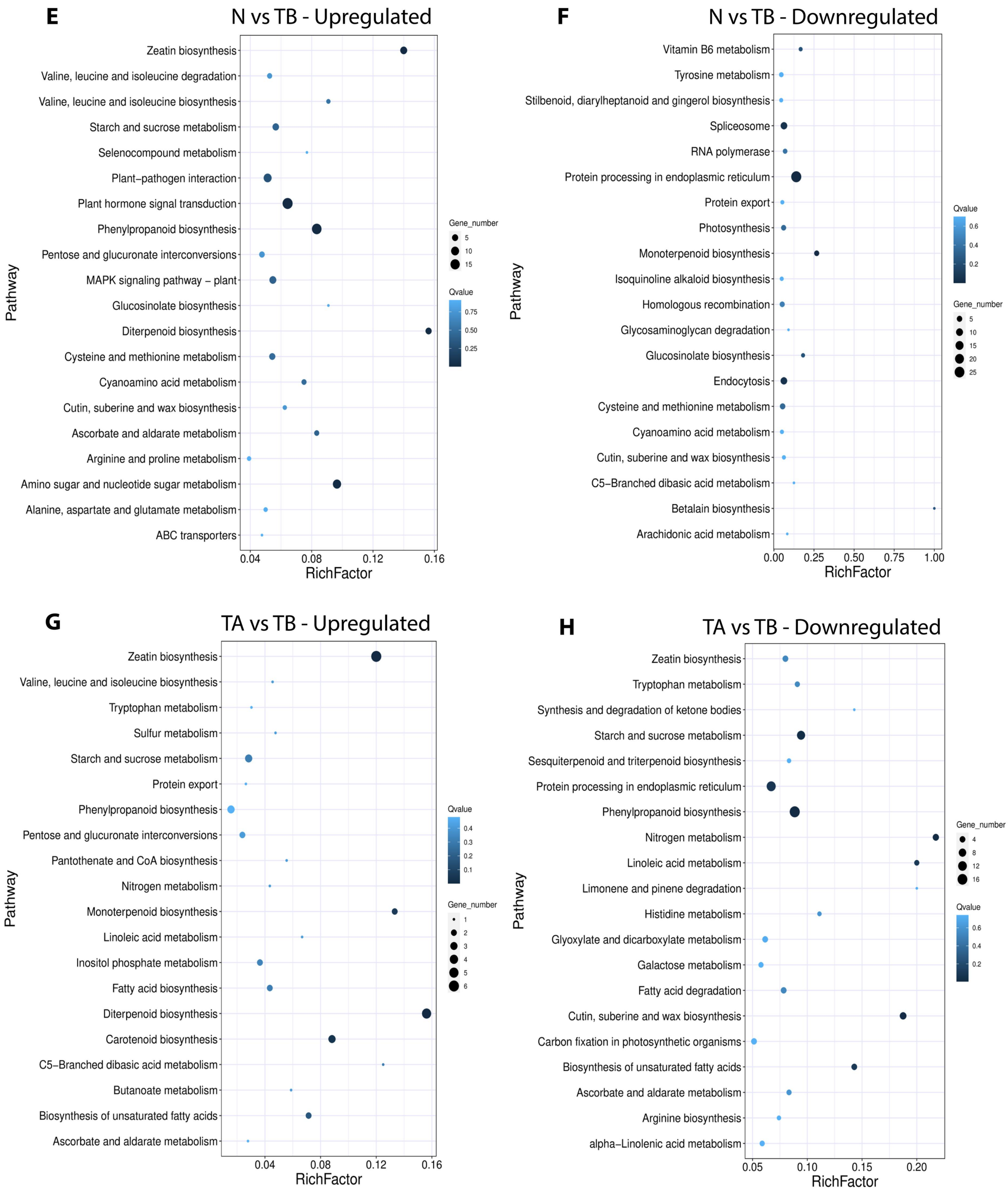

FIGURE S6 | GO and KEGG enrichment analysis of upregulated and downregulated DEGs in N versus TB and TA versus TB comparisons in *L. barbarum* shoot apices. **(A, B)** Top 30 significantly enriched GO terms across BP, MF, and CC categories for upregulated (A) and downregulated (B) DEGs in N vs. TB ($p \leqslant 0.05$). **(C, D)** Top 30 significantly enriched GO terms for upregulated (C) and downregulated (D) DEGs in TA vs. TB ($p \leqslant 0.05$). **(E, F)** Top 20 significantly enriched KEGG pathways for upregulated (E) and downregulated (F) DEGs in N vs. TB ($p \leqslant 0.05$). **(G, H)** Top 20 significantly enriched KEGG pathways for upregulated (G) and downregulated (H) DEGs in TA vs. TB ($p \leqslant 0.05$). The x-axis represents the gene ratio, the y-axis lists GO terms or KEGG pathways, dot color represents significance level (*Q*-value; dark blue: more significant; light blue: less significant), and dot size represents the number of enriched genes.

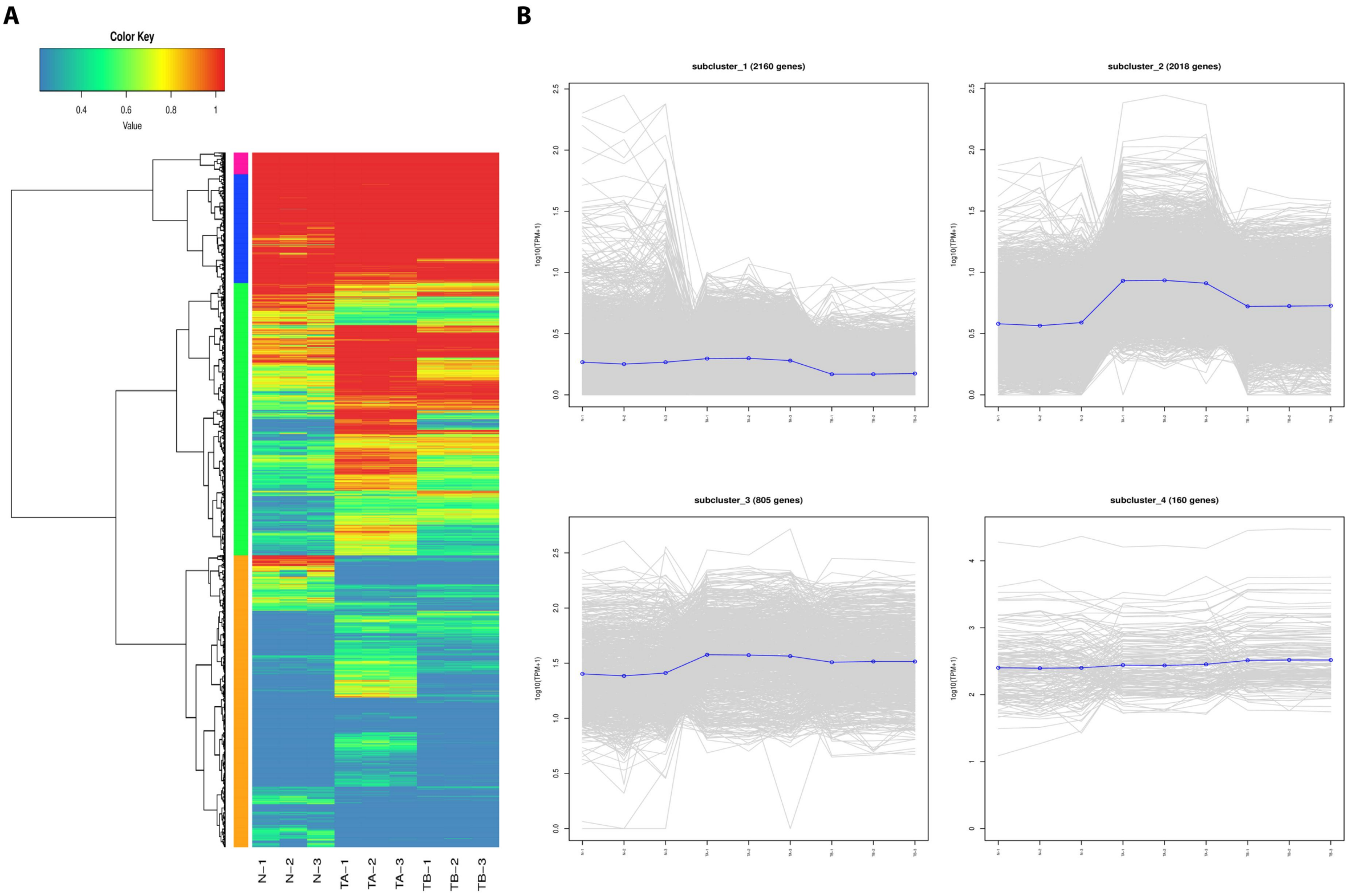

FIGURE S7 | Hierarchical clustering analysis of differentially expressed genes (DEGs) across three shoot apex sample groups in *L. barbarum*. **(A)** Heatmap showing hierarchical clustering of DEGs based on log_2-transformed TPM values across nine samples (N1-N3, TA1-TA3, and TB1-TB3), with color intensity representing relative expression levels. **(B)** Expression profile plots for four distinct DEGs subclusters identified by hierarchical clustering. Within each cluster panel, grey lines represent the relative expression trajectory of individual genes across samples, and the blue line represents the mean expression trajectory of all genes within the cluster. The x-axis represents sample groups and the y-axis represents log_{10} (TPM+1) expression values.

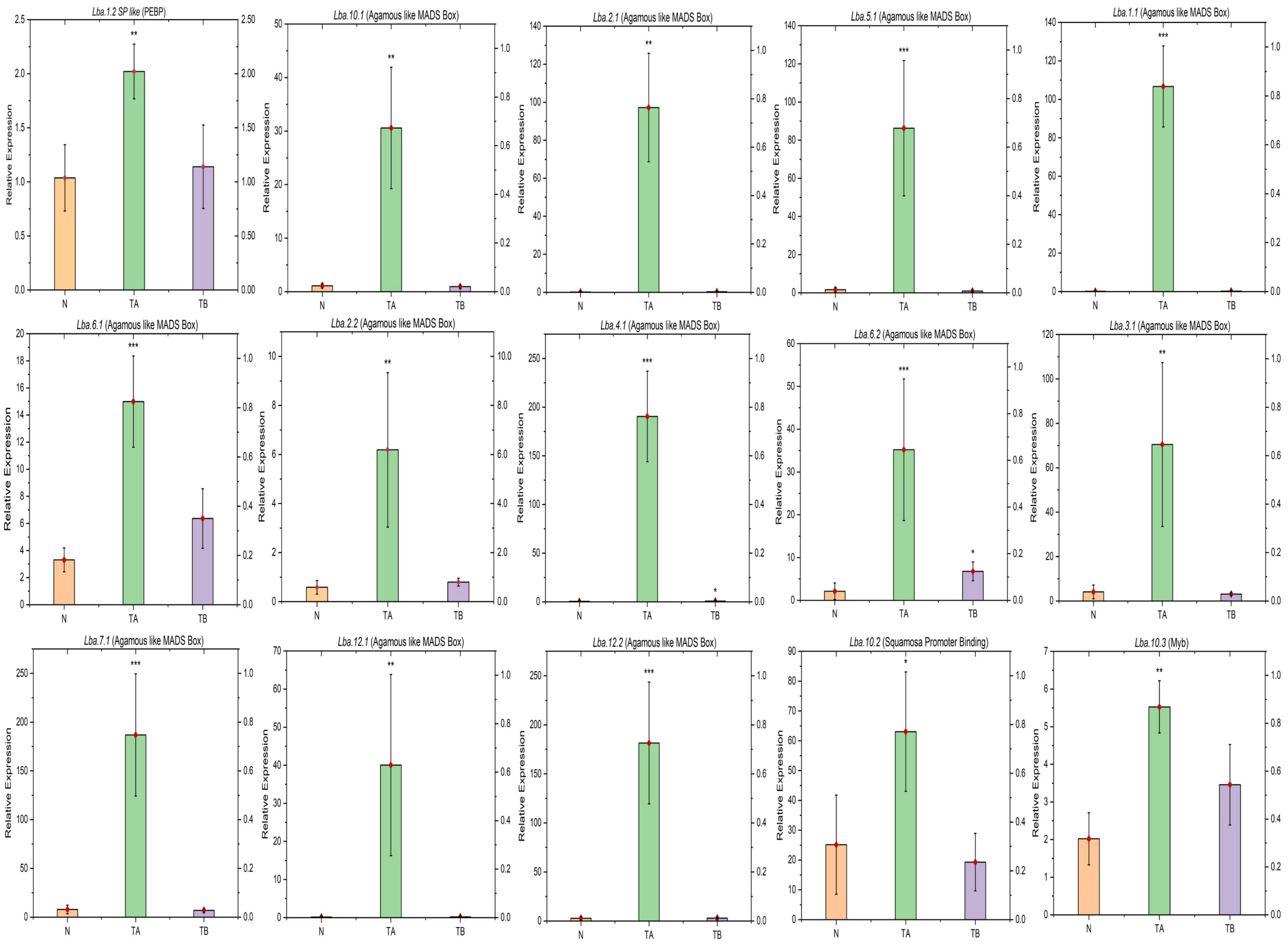

FIGURE S8 | RT-qPCR validation of fifteen candidate genes selected from transcriptome analysis of shoot apex samples (N, TA, and TB) in *L. barbarum*. Validated genes include one *SP-like* from the PEBP family (*Lba.1.2*; Lba0102749), twelve *Agamous-like* MADS-box genes (*Lba.10.1*; Lba1000776, *Lba.2.1*; Lba0200225, *Lba.5.1*; Lba0501700, *Lba.1.1*; Lba0101571, *Lba.6.1*; Lba0601235, *Lba.2.2*; Lba0201670, *Lba.4.1*; Lba0401685, *Lba.6.2*; Lba0602162, *Lba.3.1*; Lba0302512, *Lba.7.1*; Lba0701418, *Lba.12.1*; Lba1201472, *Lba.12.2*; Lba1202783), one SQUAMOSA promoter-binding protein-like (SPL) gene (*Lba.10.2*; Lba1001980), and one MYB TF gene (*Lba.10.3*; Lba1002781). *LbActin* was used as the internal control reference gene. Grouped bar graphs show relative expression levels from both RNA-seq data and RT-qPCR analysis across N, TA, and TB sample groups, with RNA-seq and RT-qPCR values presented side by side for each gene. Values are presented as means ± SD. ($n = 2$ biological replicates; Student's *t*-test; $*p < 0.05$, $**p < 0.01$, $***p < 0.001$).

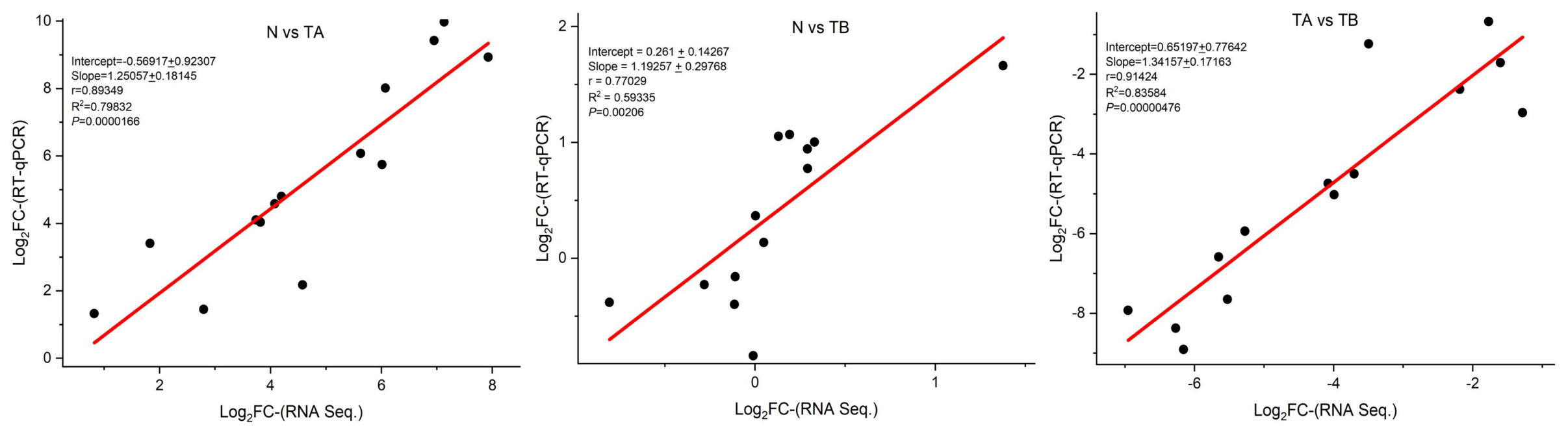

FIGURE S9 | Linear regression analysis comparing RNA-seq $\log_2$FC values and RT-qPCR $\log_2$FC values for the fifteen validated candidate genes across three pairwise comparisons (N vs. TA, N vs. TB, and TA vs. TB). Each data point represents one candidate gene. R^2 and p values indicate the Pearson correlation coefficient between RNA-seq and RT-qPCR expression values, and statistical significance of the correlation, respectively.

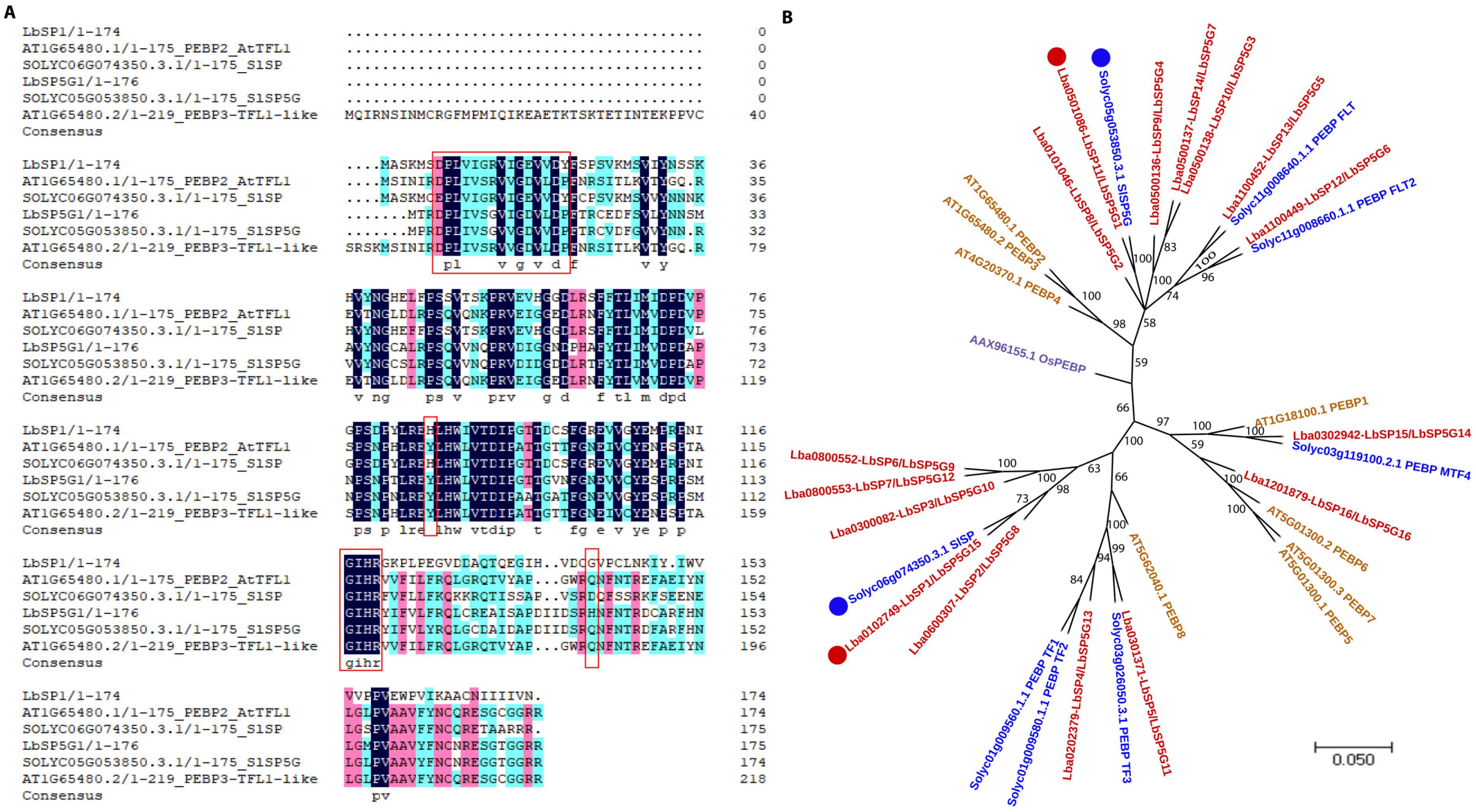

FIGURE S10 | Multiple sequence alignment and phylogenetic analysis of *LbSP1* and *LbSP5G1* with TFL1/SP family members from *Arabidopsis thaliana*, *Solanum lycopersicum*, and *Oryza sativa*. **(A)** Multiple sequence alignment of *LbSP1* (Lba0102749) and *LbSP5G1* (Lba0501086) with *SlSP* (Solyc06g074350.3.1), *SlSP5G* (*Solyc05g053850.3.1*), *AtTFL1* (*AT1G65480.1*), and *AtTFL1-like* (*AT1G65480.2*), generated using MUSCLE. Conserved residues are highlighted and the consensus sequence logo is shown below the alignment. The alignment reveals high amino acid conservation with PEBP domain—particularly at conserved motifs including DP-DP (position 50-65) and G-HR (position 160-165) critical for ligand binding and structural stability. Key residues distinguishing TFL1/SP-type repressors from FT-type activators, including histidine (His) and aspartate (Asp) substituted with tyrosine (Tyr) at position 86, and glutamine (Q) at 141, respectively, are indicated. Among the 16 *SP-like* homologues identified through BLAST searches of the *L. barbarum* genome, *LbSP1* and *LbSP5G1* exhibited low E-values and highest sequence similarity to tomato *SlSP* and *SlSP5G* respectively, and were selected for CRISPR/Cas9-mediated functional characterization. (**B**) Neighbor joining bootstrap phylogenetic tree (1000 replicates) constructed for amino acid sequences of *LbSP1*, *LbSP5G1*, and representative TFL1/SP/SP5G family members from *S. lycopersicum*, *A. thaliana*, and *O. sativa*. *LbSP1* clusters with the same clade as *SlSP* and *AtTFL1*, while *LbSP5G1* clusters with *SlSP5G*, confirming their evolutionary conservation within the TFL1/SP subfamily of the PEBP family in Solanaceae.

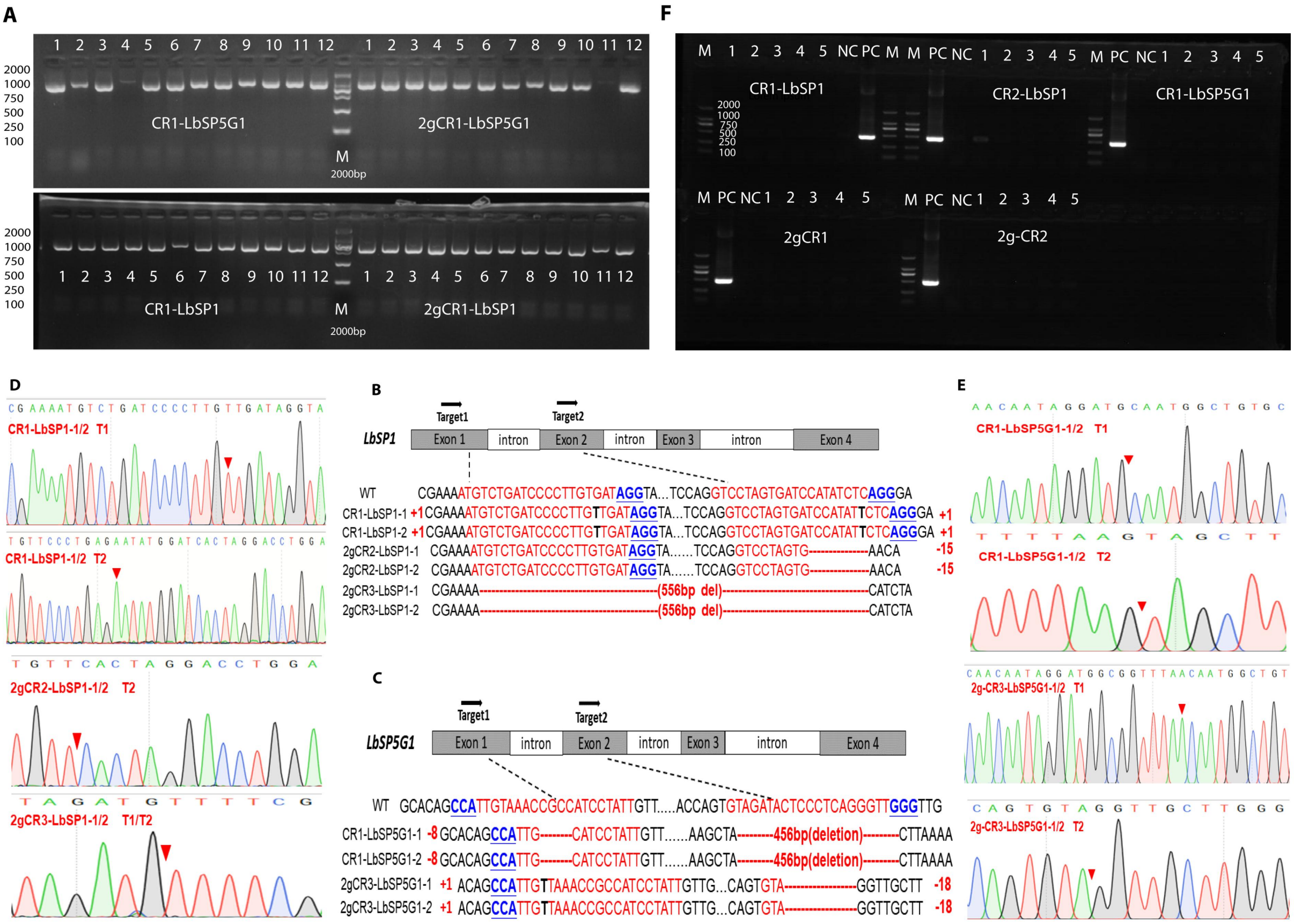

FIGURE S11 | Molecular validation of CRISPR/Cas9-mediated genome editing and transgene-free confirmation in *L. ruthenicum* edited lines. **(A)** PCR amplification using *LbSP1*- and *LbSP5G1*-specific primers flanking the respective target sites, showing expected amplicon sizes of 816bp (*LbSP1*), and 1,036bp (*LbSP5G1*) in single (CRLbSP1, CRLbSP5G1) and double (2gCRLbSP1+LbSP5G1) edited lines compared with the wild-type (WT). **(B, C)** Sanger sequencing alignment showing the two gRNA target sequences in WT and single (CR1-LbSP1, CR1-LbSP5G1) and double (2g-CR3) mutant lines for *LbSP1* (B) and *LbSP5G1* (C), with the induced mutations clearly visible at the target sites. **(D, E)** Chromatogram plots confirming homozygous mutations at the *LbSP1* (D) and *LbSP5G1* (E) target loci in single and double mutant lines. Red highlighting indicates gRNA target sequences and blue highlighting indicates PAM sites. **(F)** PCR detection of CRISPR/Cas9 vector components using Cas9 nuclease- and NPTII selectable marker-specific primers. Amplification was detected only in the positive control (CRISPR/Cas9 plasmid), while no amplification was observed in WT or any homozygous edited line, confirming successful excision of the T-DNA construct and generation of transgene-free edited plants. M, DNA molecular weight marker (2000 bp).

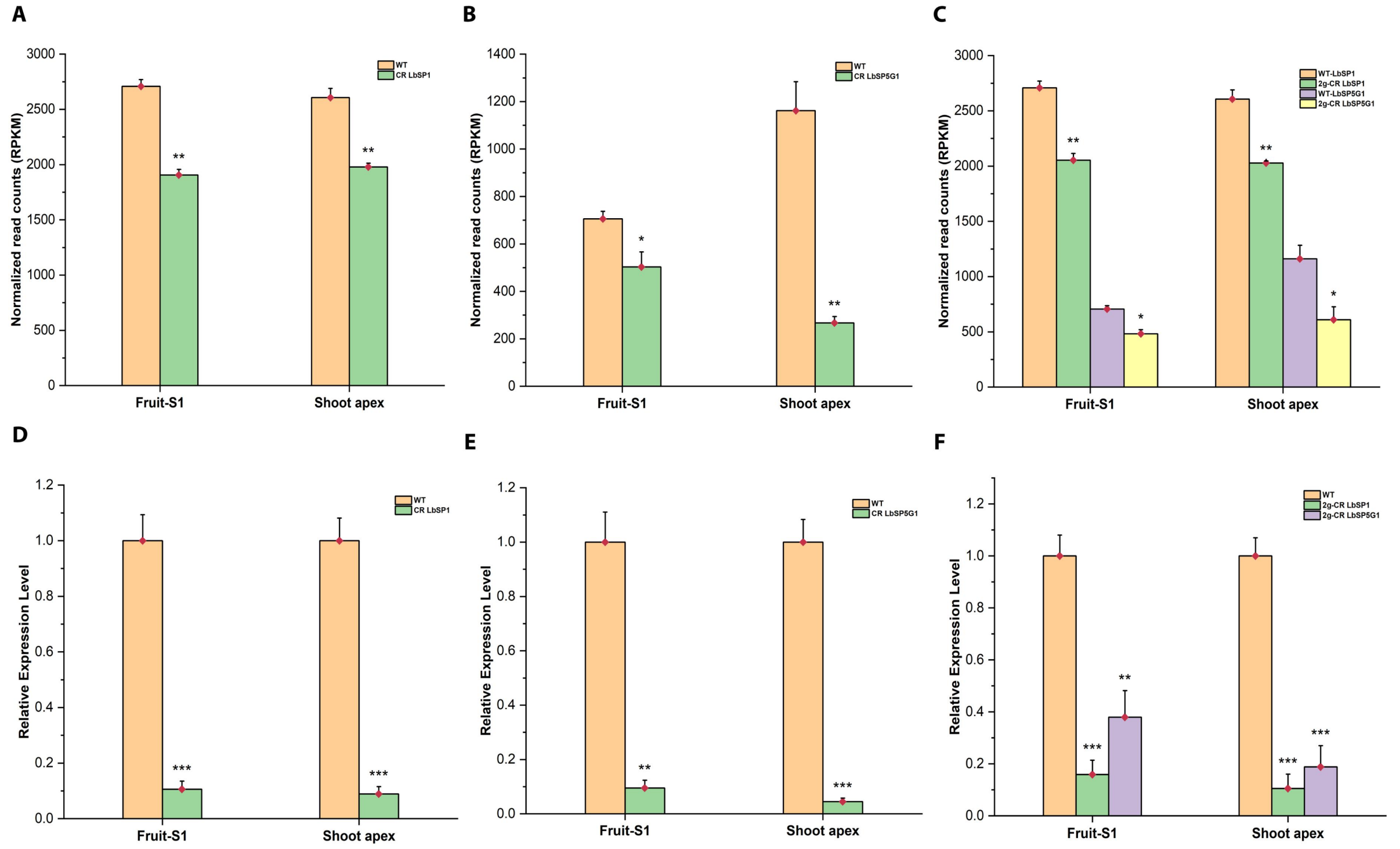

FIGURE S12 | Expression validation of *LbSP1* and *LbSP5G1* in CRISPR/Cas9-edited *L. ruthenicum* lines. **(A-C)** Normalized RNA-seq expression levels (RPKM, reads per kilobase of transcript per million mapped reads) of *LbSP1* and *LbSP5G1* in immature fruit (S1 stage), and shoot apical meristem (SAM) tissues of WT and homozygous single mutant CRLbSP1 (A), single mutant CRLbSP5G1 (B), and double mutant 2gCR LbSP1+LbSP5G1 (C) lines, demonstrating significantly reduced expression in all edited lines compared to WT. (**D-F**) RT-qPCR validation of *LbSP1* and *LbSP5G1* expression levels in S1 fruit and SAM tissues CRLbSP1 (D), CRLbSP5G1 (E), and 2gCR LbSP1+LbSP5G1 (F) edited lines compared to WT. Three independent biological replicates were analyzed for each line. Statistical significance was assessed using one-way ANOVA followed by pairwise two-tailed student's *t*-test ($p < 0.05$). Data are presented in mean ± SD.

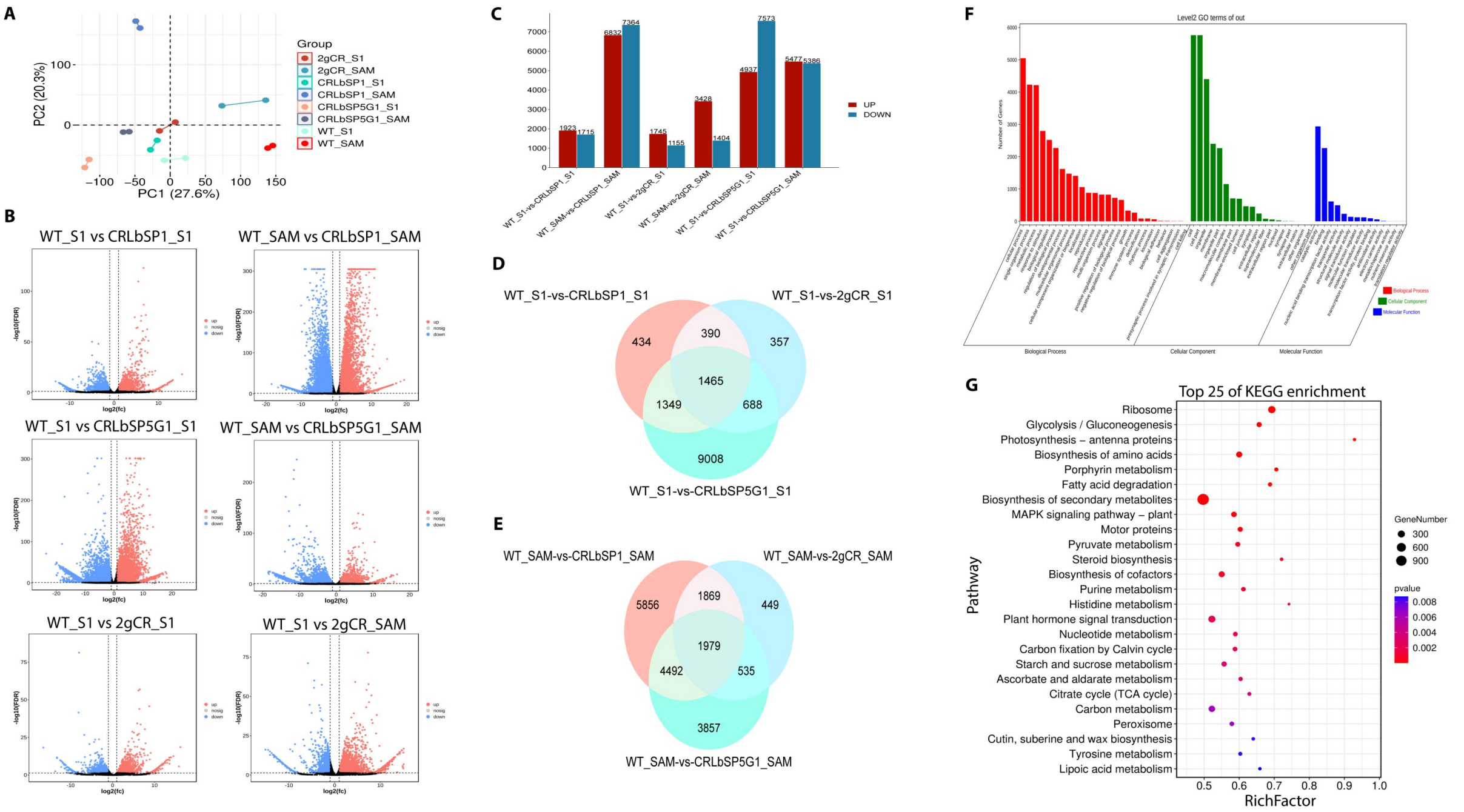

FIGURE S13 | Transcriptomic analysis of SAM and S1 fruit tissues from WT and CRISPR/Cas9-edited *L. ruthenicum* lines (CRLbSP1, CRLbSP5G1, and 2gCR LbSP1+LbSP5G1). **(A)** PCA biplot showing genotype-dependent clustering of S1 fruit and SAM samples across all four genotypes (WT, CRLbSP1, CRLbSP5G1, 2gCR), with PC1 and PC2 explaining the major sources of transcriptomic variance. **(B)** Volcano plots of DEGs in all pairwise comparisons between WT and edited lines in S1 and SAM tissues, with $\log_2$FC plotted against $-\log_{10}$ (FDR-adjusted *p*-values). Red dots: significantly upregulated DEGs; blue dots: significantly downregulated DEGs ($|\log_2FC| \geq 1$, FDR ≤ 0.05); grey dots: non-significant genes. **(C)** Bars graphs summarizing the number upregulated and downregulated DEGs in each pairwise comparisons across S1 and SAM tissues. **(D, E)** Venn diagrams showing the number of common and unique DEGs across the three pairwise comparisons in S1 fruit (D) and SAM (E) tissues. (**F, G**) GO and KEGG enrichment analysis of DEGs from all three pairwise comparisons in S1 fruit (F) and SAM (G), showing the most significantly enriched BP, MF, CC, and metabolic pathways ($p \leq 0.05$).

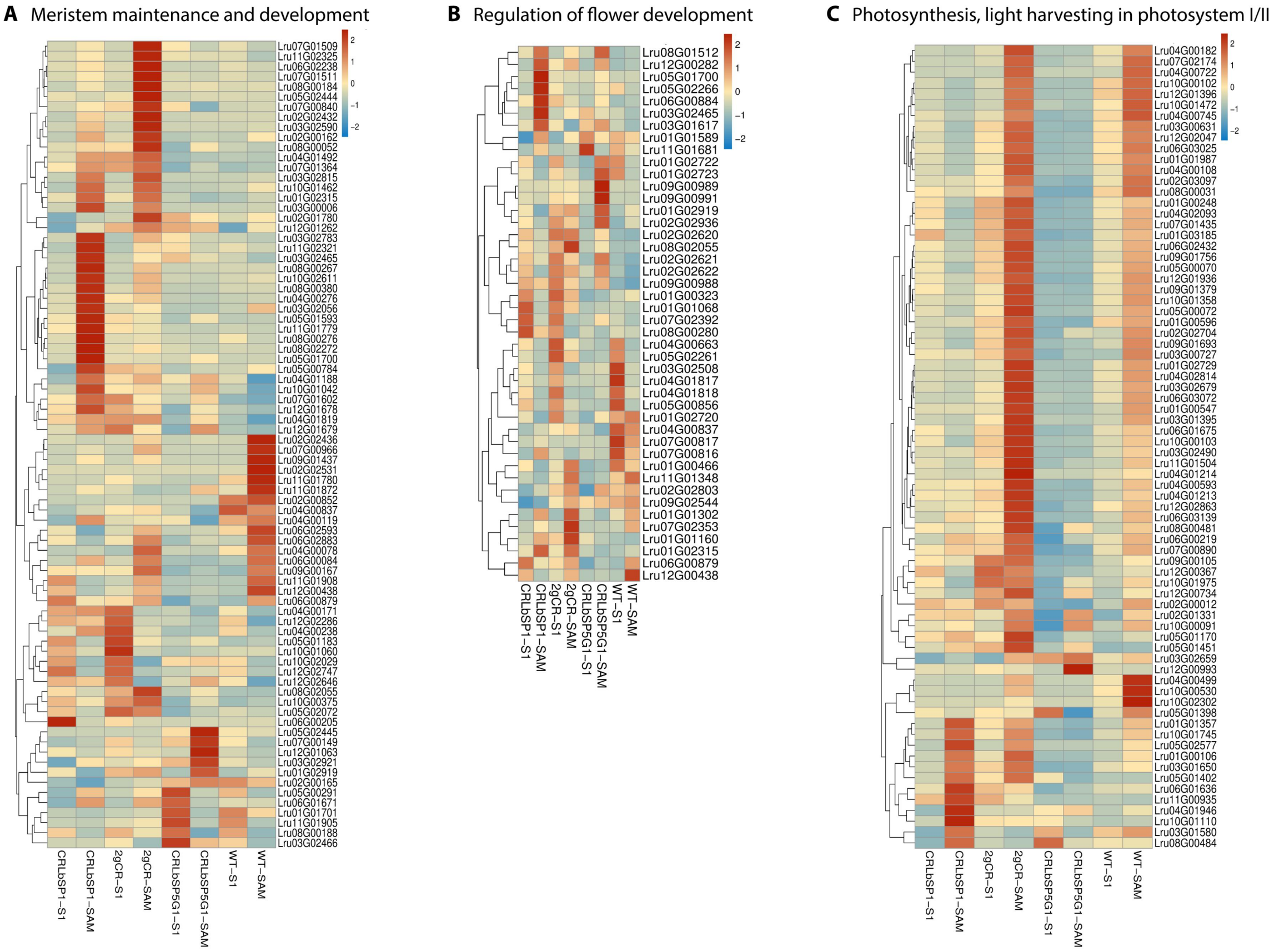

FIGURE S14 | Hierarchically clustering heatmaps showing expression profiles of GO and KEGG-enriched DEGs associated with flowering regulatory pathways in SAM and S1 fruit tissues of WT and CRISPR/Cas9-edited *L. ruthenicum* lines (CRLbSP1, CRLbSP5G1, 2gCR LbSP1+LbSP5G1). **(A)** Heatmap of DEGs associated with meristem maintenance and development, showing coordinated downregulation in edited lines relative to WT. **(B)** Heatmaps of DEGs associated with regulation of flower development, demonstrating progressive transcriptional changes from single to double edited lines. **(C)** Heatmap of DEGs associated with photosynthesis and light harvesting in photosystem I/II, showing reduced expression in edited lines with selective upregulation in the double mutant under SAM conditions. Color scale represents log_2-transformed TPM values, with red indicating high expression and blue indicating low expression.

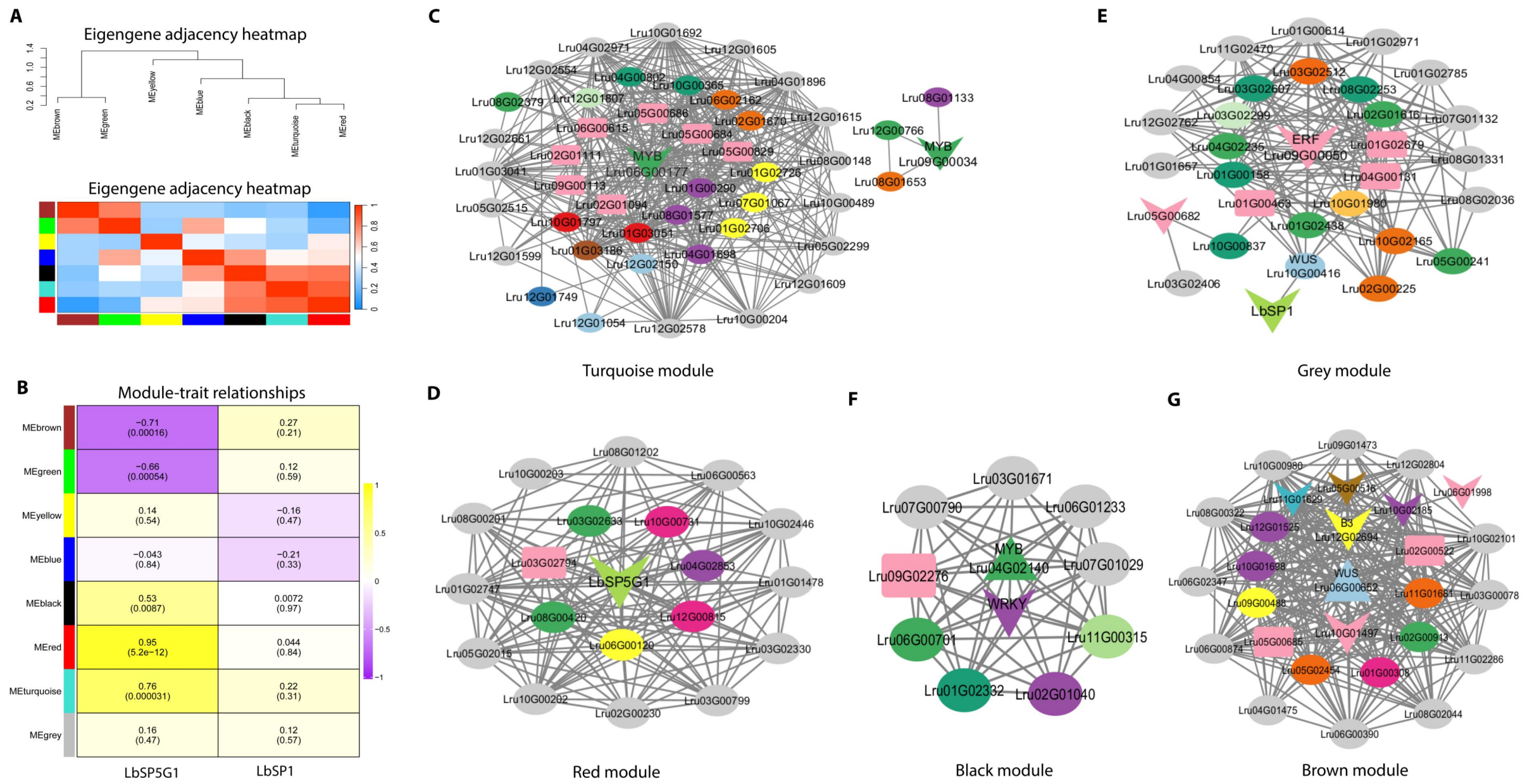

FIGURE S15 | Weighted gene co-expression network analysis (WGCNA) of DEGs from SAM and S1 fruit tissues of WT and *LbSP1/LbSP5G1*-edited *L. ruthenicum* lines. **(A)** Sample dendrogram and trait heatmap, showing hierarchical clustering of samples and module-trait correlations. **(B)** Module-trait relationship heatmap showing Pearson correlation coefficients (R) and significance values (p) between co-expression modules and *LbSP1/LbSP5G1* expression traits. Eight distinct modules were identified. **(C)** Turquoise module (394 gene; $R = 0.76$, $p = 3.1\times10^{-5}$), a tightly connected regulatory network centered on MYB transcription factor (TF) (Lru06G00177), showing strong co-expression with AP2-like/ERFs, *Agamous-like* MADS-box, WRKY, B3/ARFs, DOF zinc finger, DELLA, NAM-NAC, cup-shaped cotyledon2 (CUC), and WUS-related genes. A second MYB member (Lru09G00034) was also co-expressed with WRKY and MADS-box genes, highlighting the central regulatory significance of MYB TFs within this module. **(D)** Red module (143 genes; $R = 0.95$, $p = 5.2\times10^{-12}$)—showing the strongest positive correlation with *LbSP5G1*, with LbSP5G1 emerging as the hub gene directly co-expressed with WRKY, MYB, TCP, B3/ARFs, and AP2/ERF TFs. **(E)** Grey module (157 genes; $R = 0.12$, $p = 0.57$)—showing significant association with LbSP1, which was co-expressed with WUS and indirectly associated with ERF, NAC, and DOF TFs, suggesting involvement in meristem identity regulation. **(F)** Black module (51 genes; $R = 0.53$, $p = 0.0087$), displaying a well-defined co-expression network in which WRKY (Lru12G01412) was co-expressed with MYB (Lru04G02140), ERF, DOF, and NAC TFs, representing a direct transcriptional response to *LbSP1/LbSP5G1* knockout. **(G)** Brown module (289 genes; $R = 0.27$, $p = 0.12$), showing a negative correlation with *LbSP5G1*, and a distinct co-expression pattern involving ARFs/B3 (Lru09G00488, Lru12G02694), WUS (Lru06G00652), MADS-box, ERFs (Lru06G01998, Lru10G01497), AT-hook motif proteins (Lru05G00516), WRKY (Lru10G02185), PHR2-MYB (Lru11G01629), and TCP TFs. A TCP gene homologous to CYCLOIDEA (Lru01G00308) showed strong associations with WRKY, WUS, MADS-box, HSF, bZIP, and MYB genes, suggesting a potential role in meristem development and flowering control. Node color coding: Turquoise module, Purple: WRKY; green: MYB; orange: MADS-box; pink: ERFs; blue: bHLH; sky blue: WUS; yellow: B3 domain-containing protein or ARFs; sea green: NAC; red: DELLA; brown: CUC; light green: DOF. Red module, dark pink: TCP; bright green: *LbSP5G1*. Grey module, Amber: SPL; bright green: *LbSP1*. Brown module, olive: AT hook motif; turquoise: phosphate starvation response2 (PHR2); light grey: remaining structural genes families. Downward arrows indicate primary hub genes with the highest within-module connectivity; triangles indicate secondary hub genes with strong module membership scores.

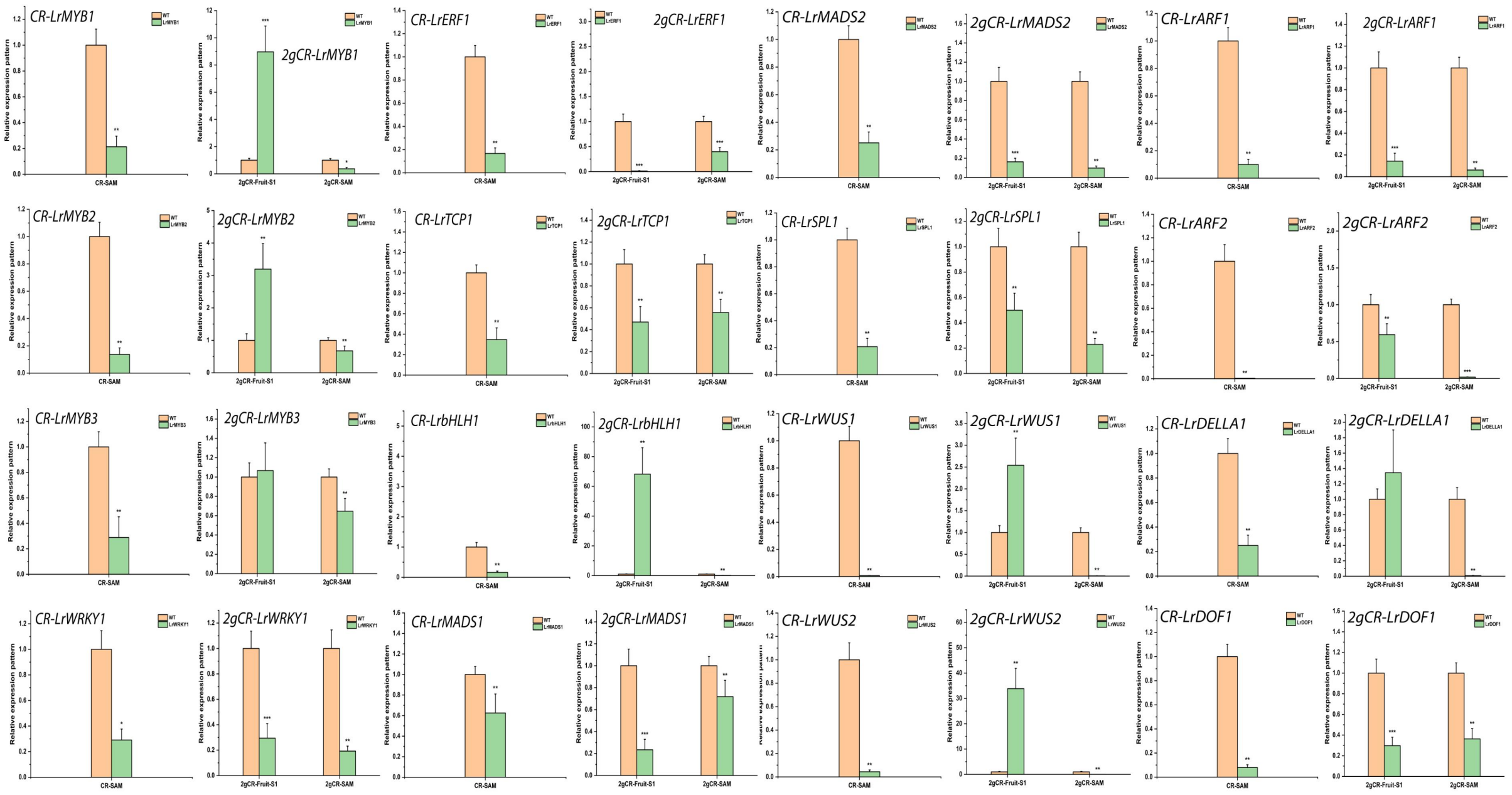

FIGURE S16 | RT-qPCR validation of co-expression network hub genes associated with flowering regulation and meristem development in SAM and S1 fruit tissues of WT and CRISPR/Cas9-edited *L. ruthenicum* lines. Expression levels of 16 candidate transcription factor genes—*LrMYB1-LrMYB3* (Lru09G00034, Lru06G00177, Lru04G02140), *LrWRKY1* (Lru12G01412), *LrERF1* (Lru09G02276), *LrTCP1* (Lru05G02299), *LrbHLH1* (Lru12G01749), *LrMADS1-LrMADS2* (Lru06G02162, Lru11G01681), *LrSPL1* (Lru10G01980), *LrWUS1-LrWUS2* (Lru10G00416, Lru12G02150), *LrARF1-LrARF2* (Lru06G00120, Lru01G02706), *LrDELLA1* (Lru01G03051), *LrDOF1* (Lru12G01807)—were measured in S1 fruit and SAM tissue of single and double CRISPR/Cas9-edited lines compared to WT. *LbActin* was used as the internal reference gene. Three independent biological replicates were analyzed for each line. Statistical significance was assessed using one way ANOVA followed by pairwise two-tailed Student's *t*-test (*$p < 0.05$, **$p < 0.01$, ***$p < 0.001$). Data are presented as mean ± SD.
